# Efficacy of different routes of triamcinolone acetonide administration on macular edema: A systematic review and network meta-analysis

**DOI:** 10.1371/journal.pone.0317782

**Published:** 2025-01-24

**Authors:** Kexin Liu, Jinyang Yi, Juan Xu, Li Zhong, Na Su

**Affiliations:** 1 Department of Pharmacy, West China Hospital, Sichuan University, Chengdu, China; 2 Department of Pharmacy, Suining First People’s Hospital, Suining, China; 3 Pharmacy Department of Zizhong County People’s Hospital, Zizhong, China; 4 Department of Clinical Pharmacy, The People’s Hospital of Zhongjiang, Zhongjiang, China; 5 West China School of Pharmacy, Sichuan University, Chengdu, China; Harvard Medical School, UNITED STATES OF AMERICA

## Abstract

There is different administration routes of triamcinolone acetonide (TA) administration for macular edema, but the efficacy ranking remains unclear. The purpose of this study is to assess the efficacy of different administration routes of TA employed in macular edema. PubMed, Medline, Embase, and Cochrane Central Register of Controlled Trials were systematically searched for published articles comparing macular edema in patients with triamcinolone acetonide in different administration. The sparse network was evaluated using a random-effects model and consistency model within the Bayesian framework, utilizing the multinma package in R. The evidence was assessed based on the Grading of Recommendations. Assessment, Development, and Evaluation (GRADE) criteria. A total of 1138 citations were identified by our search, of which 20 RCTs enrolled 892 eyes. The network showed that intravitreal triamcinolone acetonide (IVTA) was associated with a statistically significant better best corrected visual acuity (BCVA) at the 12th week compared to placebo (MD: − 0.15, 95% CI: − 0.30 to − 0.01, P < 0.05), which was moderate-quality evidence. IVTA and suprachoroidal triamcinolone acetonide (SCTA) were both associated with a statistically significant reduction in central macular thickness (CMT) at the 12th week, which was moderate evidence. The probabilities of rankings and SUCRA demonstrated that sub-Tenon’s infusion of triamcinolone acetonide (STiTA) might be the worst. SCTA and IVTA were proven to be the best administration routes for improving BCVA and reducing CMT. In addition, STiTA was less advisable than other administration routes of triamcinolone acetonide according to the rankings and SUCRA.

## Introduction

Macular edema is a consequence of various ocular and systemic disease processes, characterized by an aberrant accumulation of fluid within the macula [1]. This condition can significantly impair vision and may even lead to permanent visual loss [2]. Macular edema is most commonly observed in conjunction with diabetic retinopathy, age-related macular degeneration, retinal vascular disorders, and inflammatory diseases [3]. Out of these, diabetic retinopathy stands as the leading cause of new cases of blindness among adults aged 20–74 years in developed countries [4]. The global population consists of around 21 million individuals affected by diabetic macular edema. Among patients diagnosed with diabetes after the age of 30, there is an approximately 40% incidence rate of developing diabetic macular edema within a span of 10 years [5].

Pharmacological treatments for macular edema encompass various options, such as anti-vascular endothelial growth factor (anti-VEGF) medications, corticosteroids and so on [6–9]. Corticosteroids exhibit both anti-inflammatory and angiostatic effects, and intravitreal injection or implantation of the corticosteroids has been recommended as a second-line treatment for individuals with macular edema (DME) and retinal vein occlusion (RVO) by guidelines [4,10,11]. For certain conditions intravitreal corticosteroids might be considered as a first-line treatment [10]. Triamcinolone acetonide is one of the most commonly used corticosteroids [12]. It is the primary choice for managing macular edema. Its effectiveness in treating macular edema can be attributed to its modulation of various inflammatory factors. Additionally, its capacity to enhance diffusion through calcium channel modulation might contribute to its efficacy in reducing macular edema [13]. Different administration routes exist for triamcinolone acetonide in treating macular edema, including intravitreal triamcinolone acetonide (IVTA), suprachoroidal triamcinolone acetonide (SCTA), sub-Tenon’s infusion of triamcinolone acetonide (STiTA), orbital floor triamcinolone acetonide (OFTA) and retrobulbar injections of triamcinolone acetonide (RITA). However, only IVTA and SCTA have received recommendations for treating macular edema according to guidelines and FDA labeling [4,10,11].

Numerous randomized controlled trials (RCTs) have been conducted in the past, comparing the various administration routes of triamcinolone acetonide for treating macular edema in separate pairwise analyses. However, the definitive ranking of the efficacy among all these administration routes of triamcinolone acetonide has remained uncertain up until the present time. As a result, we initiated a network meta-analysis to comprehensively evaluate the efficacy of various administration routes of triamcinolone acetonide in the management of macular edema.

## Methods

This study was designed as a systematic review with network meta-analysis and in accordance with the items of the Preferred Reporting Items for Systematic Reviews and Meta-Analyses (PRISMA) (S1 Table) [14]. We registered the study on the International Prospective Register of Systematic Review (Registration No: CRD 42022366499).

### Literature search and eligible criteria

We comprehensively searched PubMed, MEDLINE (Ovid SP), Embase (Ovid SP), and Cochrane Central Register of Controlled Trials (Ovid SP) from inception to July 26th, 2024. In addition to the cited sources, a thorough investigation was carried out on ClinicalTrials.gov, and the reference lists of prominent reviews and meta-analyses were examined. The key terms utilized in this study were derived from the PICOS (Participants, Intervention, Comparison, Outcome, and Study design) framework, and the key terms searched included terms relating to macular edema, triamcinolone acetonide, and RCTs (S2 Table presents the full search strategy). Duplicate records were eliminated using the EndNote X9 software.

### Study selection

The studies incorporated in this analysis adhered to the subsequent criteria: 1) inclusion of adult participants (>18 years) diagnosed with macular edema; 2) interventions/comparisons: IVTA, OFTA, STiTA, RITA, SCTA or placebo; 3) outcomes: BCVA and CMT change at 12 weeks or 24 weeks from baseline. BCVA was recorded using the logarithm of the minimum angle of resolution (logMAR) visual acuity; 4) The study design included both published and unpublished randomized controlled trials (RCTs) that were restricted to the English or Chinese language. 5) The timeframe for the study required a treatment duration exceeding twelve weeks. The exclusion criteria encompassed: 1) experiments conducted on animals; 2) involvement of pregnant or lactating women as participants; 3) studies published in languages other than English or Chinese; 4) studies published solely as abstracts.

### Screening process and data extraction

Two reviewers (KL and JY) conducted an independent screening of titles and abstracts using EndNote X9 (Clarivate Analytics, Philadelphia, PA, USA), adhering to the predetermined inclusion and exclusion criteria. In cases of disagreement, a third reviewer (NS) was consulted for resolution through discussion. The data extracted encompassed various aspects such as participant characteristics, intervention details, outcomes, and study design, among others.

### Quality assessment and the certainty of evidence

The risk of bias of all included studies was evaluated by two independent reviewers (KL and JY) using the Risk of Bias Version 2 (RoB2) tool [15]. Any discrepancies were resolved through discussion with a third reviewer (NS).

### The certainty of evidence

Two reviewers, KL and JY, independently employed the GRADE approach for network meta-analysis to evaluate the level of certainty in the evidence for each outcome, categorizing it as high, moderate, low, or very low [16–18]. This assessment was made by considering factors such as bias, incoherence, inconsistency, indirectness, intransitivity, publication bias, and imprecision. In cases of disagreement, a third reviewer, NS, was consulted for resolution through discussion.

### Treatment nodes

Treatment nodes were grouped by different administration routes of triamcinolone acetonide. The common dose was defined as 4–40 mg/d for triamcinolone acetonide. Network plots were generated using the multinma package in R (version 4.1.3) [19].

### Statistical analysis

The network meta-analysis was conducted utilizing a random-effects model and consistency model within the Bayesian framework. We chose mean differences (MDs) and 95% credible intervals (CIs) for best corrected visual acuity and central macular thickness. The Markov chain Monte Carlo method was employed, utilizing four chains, with a total of 80,000 iterations following an initial burn-in period of 20,000 and a thinning factor of one. Local incoherence was evaluated, and indirect estimates were obtained through the application of node splitting models [20]. The surface under the cumulative ranking curve (SUCRA) was computed in order rank various administration routes [21].

Multiple sensitivity analyses were conducted, including the exclusion of studies involving non-diabetic macular edema, studies with a participant count below 20, studies combined with non-drug therapy, and studies with lost populations. The statistical analyses were performed using the gemtc package in R (Version 4.1.3) [22].

## Results

### Characteristics of eligible studies

**Fig 1:**
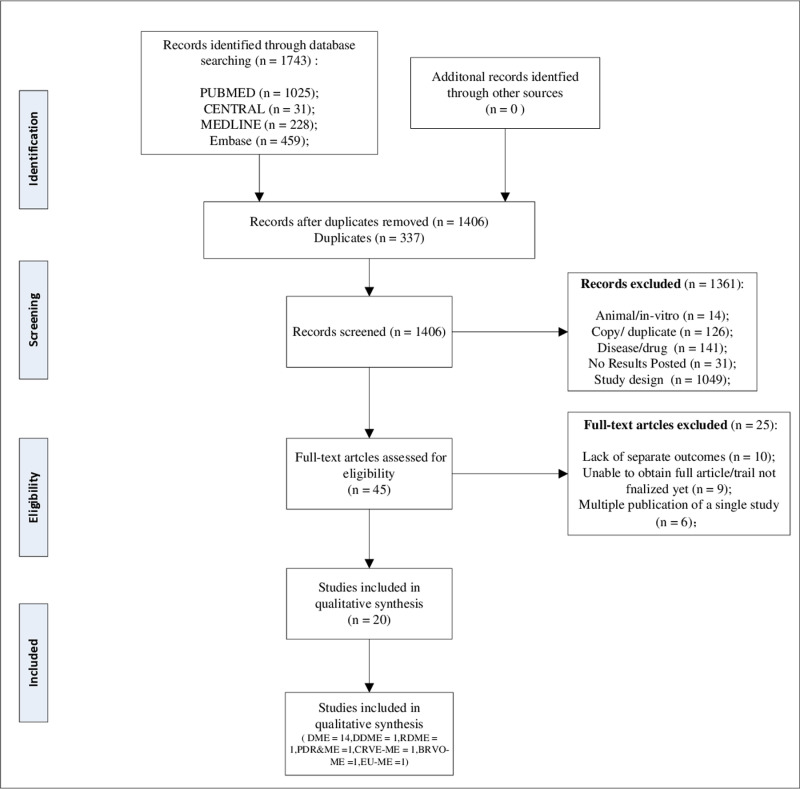
Flow diagram for study identification and inclusion. Abbreviations: DME, diabetic macular edema; DDME, diffuse diabetic macular edema; RDME, refractory diabetic macular edema; PDR&ME, proliferative diabetic retinopathy and macular edema; CRVE-ME, macular edema due to central retinal vein occlusion; BRVO-ME, macular edema associated with branch retinal vein occlusion; EU-ME, macular edema in endogenous uveitis.

The flow chart depicting the literature search process is presented in Fig 1. Following the screening of 1138 articles and registered clinical trials, a total of 20 studies conducted between 2005 and 2024 were selected for inclusion in the meta-analysis based on predetermined criteria. These studies encompassed a sample size of 786 patients, corresponding to 892 eyes [23–42]. The primary attributes of the chosen studies are compiled in Table 1. Studies were conducted in Egypt, Brazil, China, Australia, Japan, Korea, Germany, and Turkey. Among the 20 RCTs, 6 studies were registered. All the studies included in this analysis were published in the English language. The diseases examined in these studies encompassed diabetic macular edema (14 RCTs), diffuse diabetic macular edema (1RCT), refractory diabetic macular edema (1 RCT), proliferative diabetic retinopathy and macular edema (1 RCT), macular edema resulting from central retinal vein occlusion (1 RCT), macular edema associated with branch retinal vein occlusion (1 RCT), and macular edema in cases of endogenous uveitis (1 RCT). Among the studies incorporated in the analysis, 18 were classified as two-arm studies, while 2 were categorized as three-arm studies. Subsequently, we divided interventions into a placebo group and the following 5 groups: IVTA, SCTA, STiTA, RITA, and OFTA. The dosage of triamcinolone acetonide varied according to the route of administration: specifically, a dosage of 4 mg was utilized in the IVTA and SCTA groups, whereas a dosage of 40 mg was employed in the STiTA, RITA, and OFTA groups. The overall proportion of men was 51.03%. The age range of the participants spanned from 30 to 77 years. The duration of the follow-up period varied between 12 and 48 weeks. Furthermore, none of the conducted trials received financial support from pharmaceutical corporations.

**Table 1:** Characteristics baseline of randomized controlled trial.

First author	Disease	Register number/Trial name	Location	No. of eyes (n)	N	Intervention	Age	Male (%)	BCVA	CMT	Length of follow-up (weeks)
**Abdelshafy 2022**	DME	–	Egypt	23	13	SCTA 4mg	55 ± 3	6 (46.2%)	1 ± 0.05	541 ± 64	12
					10	IVTA 4mg	55 ± 5	3 (30.0%)	1 ± 0.05	512 ± 82	
**Bonini 2005**	RDME	–	Brazil	36	18	STiTA 40mg	61.48 ± 15.19	6 (42.9%)	0.9157 ± 0.1358	453.64 ± 85.956	24
					18	IVTA 4mg	62.72 ± 13.99	7 (50%)	0.9371 ± 0.1812	450.36 ± 111.5	
**Cardillo 2005**	DDME	–	Brazil	24	12	STiTA 40mg	59.0 ± 9.2	5 (41.67%)	1.077 ± 0.346	514.2 ± 195.7	24
					12	IVTA 4mg			1.077 ± 0.304	525.1 ± 177.5	
**El 2014**	DME	–	Egypt	80	40	IVTA 4mg	55.7 ± 7.2	19 (73.1%)	0.14 ± 0.12	478.10 ± 139.73	24
					40	STiTA 40mg	55.8 ± 7.9	19 (65.5%)	0.17 ± 0.11	391.82 ± 58.89	
**Feng 2010**	CRVE-ME	–	China	56	30	IVTA 4mg+RLP	60.21 ± 5.12	17 (56.67%)	0.75 ± 0.50	576 ± 114	12
					26	RLP		10 (38.46%)	0.76 ± 0.64	563 ± 120	
**Gillies 2010**	DME	NCT00148265	Australia	84	42	IVTA 4mg+Laser	65.4 ± 9.5	26 (61.9%)	–	482.1 ± 122.7	24
					42	Laser	66.9 ± 8.9	22 (52.4%)		477.4 ± 155.5	
**Hayashi 2005**	BRVO-ME	–	Japan	52	27	IVTA 4mg	65.2 ± 11.3	10 (37%)	0.598 ± 0.375	398 ± 189	24
					25	RITA 40mg	64.6 ± 10.4	10 (40%)	0.433 ± 0.285	388 ± 178	
**Lee 2009**	DME	–	Korea	60	30	MLG	59.6 ± 10.8	12 (40%)	0.53 ± 0.32	513.9 ± 55.1	24
					30	IVTA 4mg+MLG	63.6 ± 11.1	11 (36.7%)	0.61 ± 0.29	498.2 ± 19.8	
**Li 2014**	DME	–	China	64	34	RITA 20mg+PRP	53.3 ± 13.8	38 (59.38%)	0.16 ± 0.07	552.33 ± 121.32	24
					30	PLA+PRP			0.15 ± 0.08	532.00 ± 124.23	
**Luo 2014**	DME	–	China	40	20	STiTA 40mg	64.7	15 (75%)	0.814 ± 0.082	394.4 ± 21	12
					20	IVTA 4mg			0.805 ± 0.069	390.5 ± 17	
**Maia 2009**	PDR&ME	NCT00443521	Brazil	44	22	IVTA 4mg+Laser	61.9 ± 5.3	10 (45.5%)	0.44 ± 0.17	360.05 ± 84.85	48
					22	Laser			0.38 ± 0.17	331.68 ± 78.88	
**Ogura 2019**	DME	JCTNo.132139	Japan	89	27	STiTA 40mg	65.8 ± 8.23	17 (63.0%)	–	487.4 ± 141.92	12
					31	PLA	65.5 ± 7.87	22 (71.0%)		460.1 ± 108.80	
**Roesel 2009**	EU	NCT00403832	Germany	40	20	IVTA 4mg	50.0 ± 20.0	6 (30%)	0.76 ± 0.41	384.8 ± 149.4	24
					20	OFTA 40mg	54.8 ± 16.8	9 (45%)	0.74 ± 0.43	263.5 ± 139.3	
**Saleh 2017**	DME	–	Egypt	34	17	IVTA 4mg	52.53 ± 1.8	7 (41.2%)	0.865 ± 0.389	–	24
					17	STiTA 40mg	56.35 ± 2.9	8 (47.1%)	0.929 ± 0.224		
**Soliman 2018**	DME	–	Egypt	30	15	IVTA 4mg	57.9 + 7.2	7 (46.67%)	0.19 ± 0.14	427.07 ± 114.21	12
					15	STiTA 40mg	59.4 + 2.8	6 (40%)	0.25 ± 0.12	395.67 ± 60.94	
**Takata 2010**	DME	–	Brazil	19	10	IVTA 4mg	66.7 ± 5.1	6 (60%)	–	474.1 ± 134.08	24
					9	STiTA 40mg	60.8 ± 10.4	4 (44.44%)		490.8 ± 212.4	
**Wickremasinghe 2008**	DME	NCT00148330	Australia	28	13	IVTA 4mg	61.8 ± 10.9	5 (38.5%)	–	426.0 ± 146.7	12
					15	PLA	65.1 ± 11.2	7 (46.7%)		475.3 ± 98.3	
**Xu 2014**	DME	–	China	62	31	IVTA 4mg	51.3 ± 3.1	36 (58.06%)	0.12 ± 0.04	514.73 ± 56.13	36
					31	RITA 40mg			0.13 ± 0.05	509.68 ± 55.78	
**Yalcinbayir 2011**	DME	–	Turkey	28	14	IVTA 4mg	59.6 ± 8.6	9 (64.29%)	0.8643 ± 0.2977	–	24
					14	STiTA 40mg			0.7071 ± 0.3474		
**Zakaria 2022**	DME	NCT04069780	Egypt	43	15	IVTA 4mg	57.67 ± 6.62	4 (33.3%)	0.59 ± 0.25	443.33 ± 112.82	24
					15	SCTA 4mg	57.54 ± 6.33	2 (15.4%)	0.73 ± 0.28	495.80 ± 143.23	

Abbreviations: RDME, refractory diabetic macular edema; DDME, diffuse diabetic macular edema; BRVO-ME, macular edema associated with branch retinal vein occlusion; DME, diabetic macular edema; PDR&ME, proliferative diabetic retinopathy and macular edema; EU-ME, macular edema in endogenous uveitis; CRVE-ME, macular edema due to central retinal vein occlusion; RLP, retinal laser photocoagulation; MLG, macular laser grid; PRP, panretinal photocoagulation.

### Risk of bias of included studies

**Fig 2:**
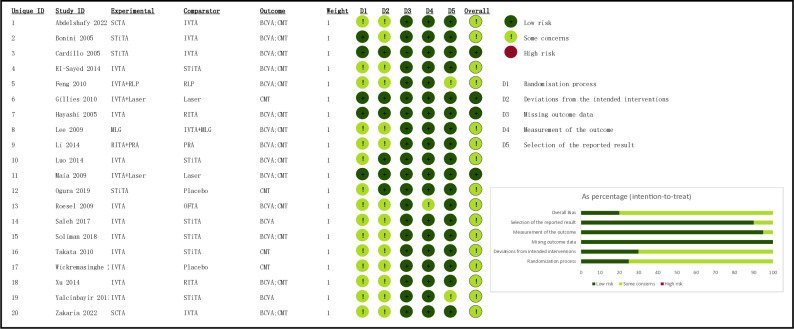
Summary of the risk of bias. Footnotes: D1: Risk of bias arising from the randomization process; D2: Risk of bias due to deviations from the intended interventions; D3: Risk of bias due to missing outcome data; D4: Risk of bias in measurement of the outcome; D5: Risk of bias in selection of the reported result; Overall: Overall risk of bias.

The risk of bias for the 20 included RCTs were assessed using Cochrane’s RoB2 tool. The overall risk of bias was deemed to be not high due to certain limitations observed in the study. These limitations primarily included the absence of comprehensive details regarding randomization methods and the relatively low level of reported blinding of participants. It should be noted that blinding was challenging in this case as triamcinolone acetonide was administered through injection, making it impossible to conceal the treatment allocation. In relation to the BCVA and CMT outcomes, which were objectively assessed and remained uninfluenced by evaluators, the major of the randomized controlled trials (RCTs) exhibited a low risk in terms of outcome measurement and selection of reported results in this analysis. The evaluation of bias risk in the studies incorporated is depicted in Fig 2. Out of the total number of studies, four (20%) exhibited a low risk of bias, while sixteen (80%) displayed certain some concerns of bias. None of the trials were found to possess a high risk of bias. Based on the similarity in study design, outcome measures, patient demographics, and inclusion and exclusion criteria, a network meta-analysis was considered suitable for conducting a quantitative synthesis of the evidence. The confirmation of homogeneity and consistency assumptions further supported this approach.

### Results of network meta-analysis

**Fig 3:**
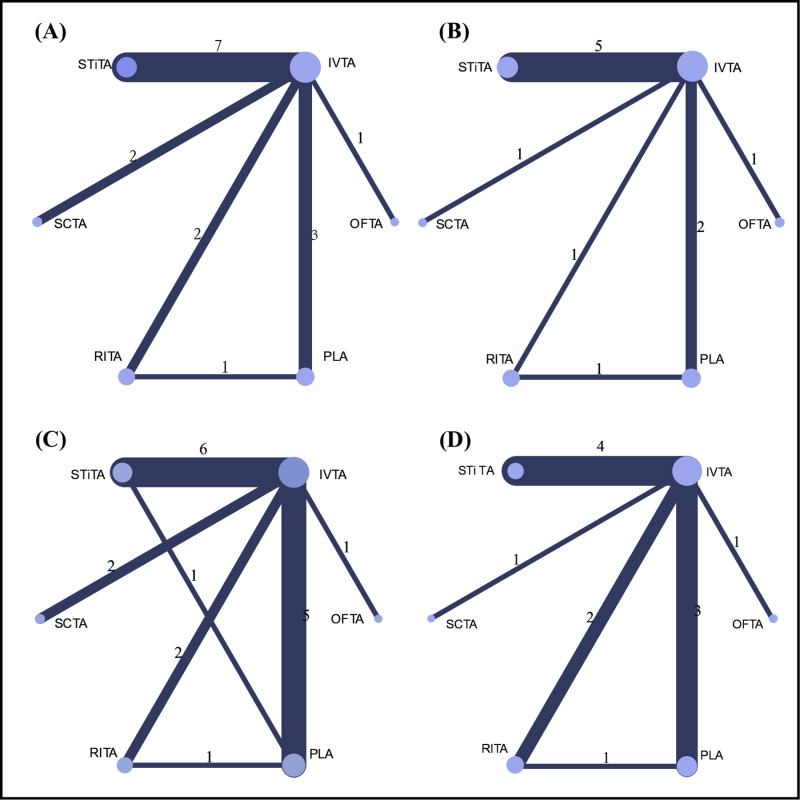
Network plots of available direct comparisons. Footnotes: BCVA at the 12th (A) and 24th (B) weeks. CMT at the 12th (C) and 24th (D) weeks. Each node (solid circle) stands for a different administration route of triamcinolone acetonide. The size of the nodes is proportional to the number of participants (i. e., sample size) involving the specific treatment intervention. The solid lines link treatments with direct comparison with the thickness proportional to the number of trials.

The network diagrams, which showcase each outcome, are visually represented in Fig 3A–3D. The assessment of the heterogeneity and inconsistency of the network meta-analysis are also conducted in S1–S8 Figs. The detailed results of evidence for all comparisons and outcomes in the network meta-analysis are presented in S4–S7 Tables. The results of the sensitivity analysis are presented in S12–S25 Tables.

### BCVA at the 12th week

**Fig 4:**
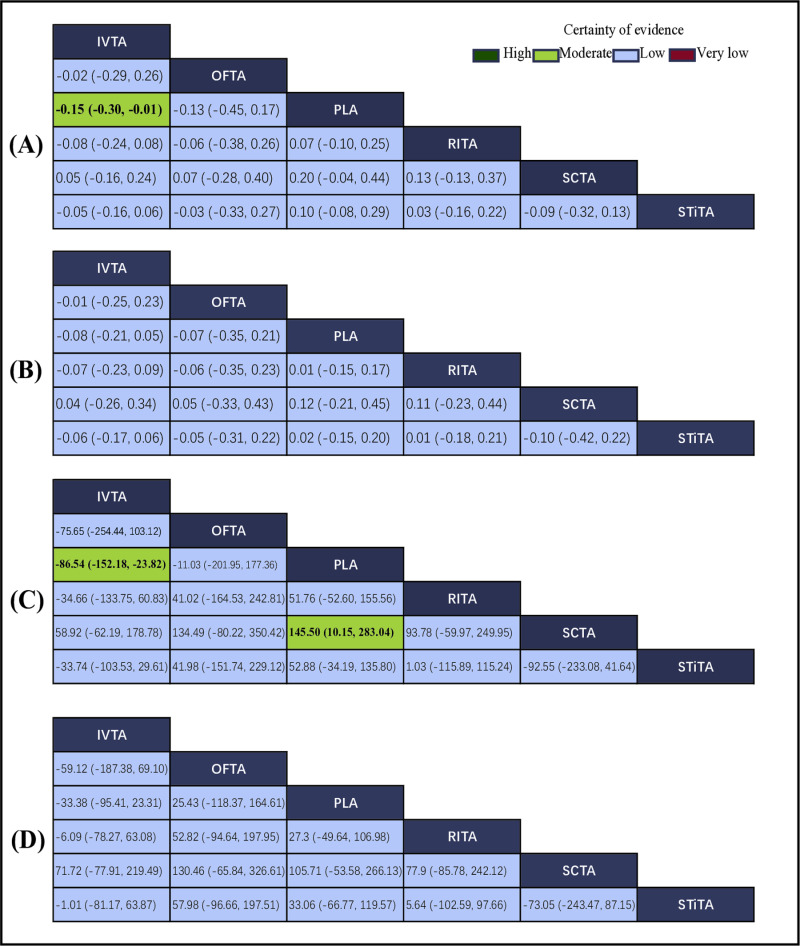
League tables of outcome analyses. **Footnotes:** BCVA at the 12th (A) and 24th (B) weeks. CMT at the 12th (C) and 24th (D) weeks. Bold indicates statistical significance. The color of each cell indicates the certainty of evidence according to the Grading of Recommendations Assessment, Development, and Evaluation.

Sixteen RCTs including 703 eyes reported BCVA after 12 weeks of different routes of triamcinolone acetonide administration on macular edema. The intervention nodes incorporated in this network meta-analysis encompassed IVTA, STiTA, OFTA, RITA, SCTA and placebo. Compared to placebo, IVTA was associated with a statistically significant better BCVA at the 12th week [MD: − 0.15, 95% CI: − 0.30 to − 0.01, P < 0.05] (Fig 4A), which was moderate-quality evidence. The global index of agreement (I²) for pairwise comparisons was found to be 0%. No statistically significant variations were observed in the remaining pairwise comparisons, and the certainty of evidence was low for other comparisons. Comprehensive data pertaining to these comparisons can be found in the appendix (S1, S5 Figs and S4 Table).

### BCVA at the 24th week

Eleven RCTs including 502 eyes reported BCVA after 24 weeks of different routes of triamcinolone acetonide administration on macular edema. The intervention nodes incorporated in this network meta-analysis encompassed IVTA, STiTA, OFTA, RITA, SCTA and placebo. There were no significant differences in either pairwise or network estimates (Fig 4B). The level of certainty in the evidence was deemed to be low across all comparisons (Fig 4B). Comprehensive data pertaining to these comparisons can be found in the appendix (S2, S6 Figs and S5 Table).

### CMT at the 12th week

Eighteen RCTs including 821 eyes reported CMT after 12 weeks of different routes of triamcinolone acetonide administration on macular edema. The intervention nodes incorporated in this network meta-analysis encompassed IVTA, STiTA, OFTA, RITA, SCTA and placebo. Compared to placebo, IVTA was associated with a statistically significant reduction in CMT at the 12th week [MD: −86.54, 95% CI: −152.18 to −23.82, P < 0.05] (Fig 4C), which was moderate evidence. The global I² of pairwise was 94.8%. SCTA was associated with a statistically significant better CMT at the 12th week [MD: −145.5, 95% CI: −283.04 to −10.15, P < 0.05] (Fig 4C), which was moderate evidence. No substantial disparities were observed in the remaining pairwise comparisons, and the level of certainty regarding the evidence for these comparisons was deemed to be low. Comprehensive data pertaining to these comparisons can be found in the appendix (S3, S7 Figs and S6 Table).

### CMT at the 24th week

Twelve RCTs including 592 eyes reported CMT after 24 weeks of different routes of triamcinolone acetonide administration on macular edema. The intervention nodes incorporated in this network meta-analysis encompassed IVTA, STiTA, OFTA, RITA, SCTA and placebo. There were no significant differences in either pairwise or network estimates (Fig 4D). The level of certainty in the evidence was deemed to be low across all comparisons (Fig 4D). Comprehensive data pertaining to these comparisons can be found in the appendix (S4, S8 Figs and S7 Table).

### Rankings and SUCRA

**Fig 5:**
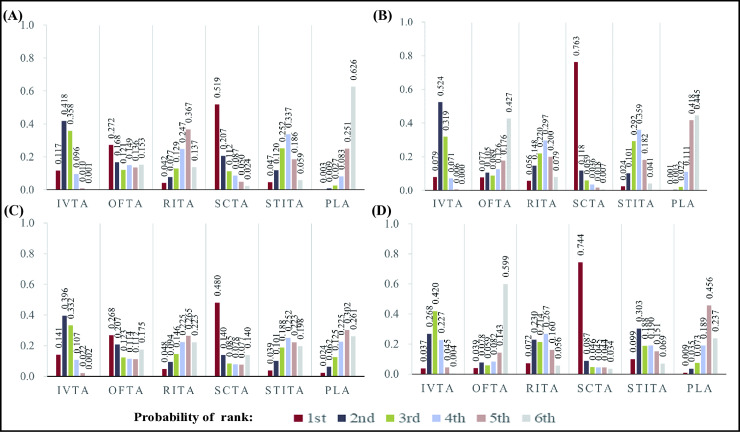
Rank diagrams of outcome analyses. BCVA at the 12th (A) and 24th (B) weeks. CMT at the 12th (C) and 24th (D) weeks. The numbers on the vertical axis represent the probability.

The rank probabilities of different routes of triamcinolone acetonide administration and placebo are depicted in Fig 5. The SUCRA values of CMT and BCVA at the 12th week for different administration routes of triamcinolone acetonide are illustrated in S8, S10 Tables, and the SUCRA values of CMT and BCVA at the 24th week are illustrated in S9, S11 Tables. The higher SUCRA value indicated the higher efficiency of BCVA and CMT. S8 and S9 Tables showed that the highest SUCRA value of BCVA at the 12th week was SCTA (SUCRA value = 0.7970) and BCVA at the 24th week was IVTA (SUCRA value = 0.7042). S10 and S11 Tables show that the highest SUCRA value of CMT either at the 12th week or 24th week were also SCTA (SUCRA value = 0.9093 or 0.8685). Whether improving BCVA or reducing CMT, the lowest SUCRA value was STiTA.

### Sensitivity analyses

The sensitivity analyses are displayed in S12–S25 Tables, showcasing their alignment with the observed outcomes.

## Discussion

Our network meta-analysis provides an overview of the evidence evaluating the efficacy of different administration routes of triamcinolone acetonide for macular edema. IVTA showed significant differences compared to placebo in improving BCVA and reducing CMT at 12 weeks. SCTA showed significant difference in reducing CMT compared with placebo at 12 weeks. There was no significant difference in the efficacy of BCVA or CMT between the different administration routes of triamcinolone acetonide with very low-to-moderate certainty at 24 weeks. According to the results of ranking and SUCRA, we find that IVTA ranked the first in BCVA and SCTA ranked the first in CMT at 24 weeks. Meanwhile, SCTA ranked the first in CMT at 12 weeks. Therefore, SCTA showed the advantage in CMT whether at 12 weeks or at 24 weeks. In addition, the doses of triamcinolone acetonide is different depending on the different administration routes.

Administering triamcinolone acetonide to the suprachoroidal space could potentially reduce the risk of specific adverse events (AEs) linked to drug exposure in the anterior chamber [43], there is still a need for further evaluation of its treatment effectiveness. Our randomized models, globally including 892 eyes, were sufficiently powered to detect statistically significant differences between the study and control groups in terms of CMT but not in BCVA. In fact, The approval of triamcinolone acetonide injectable suspension by the FDA for the treatment of macular edema associated with uveitis was predicated on the results obtained from the phase 3 PEACHTREE study (NCT02595398) [44], Post hoc analyses of the PEACHTREE data revealed a statistically significant (p <  0.001) disparity in the proportion of patients who experienced improvement in best-corrected visual acuity (BCVA) from baseline at the 24-week mark, with a larger proportion observed in the SCTA group compared to the sham group [45,46]. Although there were slight discrepancies between the results reported in the literature and our network meta-analysis, our data appear to be slightly undersized. As we included in our network meta-analysis the logMAR visual acuity as the outcome of BCVA measure, PEACHTREE was excluded because it specified early treatment diabetic retinopathy study (ETDRS) letters as the outcome of BCVA measure. However, in our opinion, the selection criteria applied in our meta-analysis are the first and foremost ones. Some recent studies report that SCTA is useful and applicable for macular edema at week 12 and through week 24 [47,48]. The rank probabilities of various routes of administration for triamcinolone acetonide and placebo are noteworthy. At the 12th week, in terms of CMT and BCVA, the top SUCRA value for triamcinolone acetonide treatment among different routes of administration was observed with SCTA. Based on the ranking probabilities, it can be inferred that SCTA may exhibit the highest effectiveness.

IVTA were the earliest routes of administration of triamcinolone acetonide in the treatment of ME [49–51]. According to the European Society of Retina Specialists (EURETINA) and the American Diabetes Association (ADA) guidelines, IVTA of corticosteroids was recommended as a second choice for treating ME [4,11]. The latest research shows that IVTA might be effective in persistent or frequent recurrent DME after anti-VEGF therapy [52]. Over recent years, several studies have shown the efficacy of IVTA injections in patients with DME and others. Our results are substantially in accordance with these studies, confirming that the effect of IVTA is rather durable, lasting more than 12 weeks and observing a drop in outcomes at the 24th week as medical literature usually reports [53–55]. Of note, another meta-analysis from Gao et al. showed that IVTA could improve BCVA in both the short and long term in patients with DME, and the long term here means that IVTA is still effective in improving BCVA at 24 weeks [56]. Although this is different from our results, this difference may be because the IVTA in Gao et al emphasized higher dosages of triamcinolone acetonide ( ≥ 8 mg), while we analyzed conventional dosages of triamcinolone acetonide (4 mg).

Earlier studies have shown that IVTA may be more effective than STiTA for the management of RDME and DME [24,25,36]. Recent studies have confirmed the efficacy of STiTA compared to placebo in the treatment of DME [34]. However, our analysis demonstrated no true superiority of STiTA with regard to the reduction in CMT and improvement in BCVA. Similarly, RITA and OFTA, had a lower probability of being the best treatment.

In this study, we examined the efficacy of various drug delivery routes based on current evidence. Additionally, it is imperative to consider the safety profiles of these drugs when administered through different routes in clinical practice. Research has indicated that the primary adverse effects associated with IVTA include ocular hypertension, cataract formation, and infectious or sterile endophthalmitis [57]. SCTA doesn’t penetrate the eye and affects fewer anatomical structures, implying a low risk of glaucoma and cataract, but increased intraocular pressure remains a concern [58]. Recent research indicates that STiTA, while less effective than IVTA in managing uveal macular edema, offers benefits in terms of substantially lower cost and decreased risk of post-injection ocular hypertension [59]. Maggio et al found that RITA was proposed as a treatment with fewer side effects than IVTA, such as a lower risk of steroid-induced cataracts, intraocular pressure increase, and no risk of endophthalmitis or rhegmatogenous retinal detachment [60]. These findings indicate that while IVTA and SCTA currently offer the best therapeutic effects, their adverse effects, particularly increased intraocular pressure, require careful monitoring.

Current systematic reviews on the utilization of triamcinolone acetonide for treating macular edema have largely been confined to meta-analyses, predominantly comparing it either to placebo alone or to anti-VEGF treatments [61–63]. Our review stands out due to its notable strengths, which encompass the most comprehensive aggregation of evidence up to the present time regarding the advantages of triamcinolone acetonide via diverse administration routes for macular edema treatment, incorporating the latest research publications. As far as we know, this represents the pioneering network meta-analysis to evaluate and contrast the efficacy of triamcinolone acetonide across its various administration routes for addressing macular edema. Our results on laboratory outcomes are consistent with a previous meta-analysis that explored the effect of BCVA improvement and CMT reduction [64]. We employed advanced methodologies to classify and present the results utilizing GRADE frameworks.

The review is subject to certain limitations, primarily stemming from the restricted quality of evidence, likely attributable to the limited number of RCTs. Consequently, this has had an impact on the validity of indirect comparisons for certain network estimates. However, the inclusion of additional high-quality RCTs would effectively address this issue. The second limitation pertains to the lack of stringent inclusion criteria for diseases. Our network meta-analysis encompassed macular edema resulting from diverse conditions, including diabetes, central retinal vein occlusion, branch retinal vein occlusion, and endogenous uveitis. The third constraint pertains to the limited sample size observed in certain RCTs that were incorporated into the current study. Nevertheless, our sensitivity analyses indicated that there were no significant disparities in the outcomes when we excluded studies involving non-diabetic macular edema, combined non-pharmacological interventions, trials with fewer than 20 participants, and studies with lost populations.

## Conclusion

This network meta-analysis suggested that SCTA and IVTA were proven to be the best administration routes for improving BCVA and reducing CMT at the 12th week in treatment for macular edema. Triamcinolone acetonide administration via different routes and placebo were no significant differences in either pairwise or network estimates at the 24th week. However, given that the SUCRA values of the different administration routes of triamcinolone acetonide was higher than placebo, triamcinolone acetonide still be cautiously used recommended for the treatment of macular edema. Furthermore, it is better to avoid the use of STiTA compared with other administration routes of triamcinolone acetonide based on the rankings and SUCRA. More large-scale multi-center randomized controlled trials are needed due to the limited number of articles available for the study.

## Supporting Information

S1 Fig Heterogeneity assessments for BCVA at the 12th week of triamcinolone acetonide treatment by different routes of administration. **Footnote:** OFTA: Orbital floor triamcinolone; IVTA: Intravitreal injection triamcinolone; RITA: Retrobulbar injections triamcinolone; SCTA: Suprachoroidal triamcinolone; STiTA: Sub-Tenon’s infusion of triamcinolone; PLA: Placebo; BCVA: Best corrected visual acuity.
(TIF)

S2 Fig Heterogeneity assessments for BCVA at the 24th week of triamcinolone acetonide treatment by different routes of administration. **Footnote:** OFTA: Orbital floor triamcinolone; IVTA: Intravitreal injection triamcinolone; RITA: Retrobulbar injections triamcinolone; SCTA: Suprachoroidal triamcinolone; STiTA: Sub-Tenon’s infusion of triamcinolone; PLA: Placebo; BCVA: Best corrected visual acuity.
(TIF)

S3 Fig Heterogeneity assessments for CMT at the 12th week of triamcinolone acetonide treatment by different routes of administration. **Footnote:** OFTA: Orbital floor triamcinolone; IVTA: Intravitreal injection triamcinolone; RITA: Retrobulbar injections triamcinolone; SCTA: Suprachoroidal triamcinolone; STiTA: Sub-Tenon’s infusion of triamcinolone; PLA: Placebo; CMT: Central macular thickness.
(TIF)

S4 Fig Heterogeneity assessments for CMT at the 24th week of triamcinolone acetonide treatment by different routes of administration. **Footnote:** OFTA: Orbital floor triamcinolone; IVTA: Intravitreal injection triamcinolone; RITA: Retrobulbar injections triamcinolone; SCTA: Suprachoroidal triamcinolone; STiTA: Sub-Tenon’s infusion of triamcinolone; PLA: Placebo; CMT: Central macular thickness.
(TIF)

S5 Fig Inconsistency (incoherence) assessments for BCVA at the 12th week of triamcinolone acetonide treatment by different routes of administration. **Footnote:** IVTA: Intravitreal injection triamcinolone; RITA: Retrobulbar injections triamcinolone; PLA: Placebo; BCVA: Best corrected visual acuity.
(TIF)

S6 Fig Inconsistency (incoherence) assessments for BCVA at the 24th week of triamcinolone acetonide treatment by different routes of administration. **Footnote:** IVTA: Intravitreal injection triamcinolone; RITA: Retrobulbar injections triamcinolone; PLA: Placebo; BCVA: Best corrected visual acuity.
(TIF)

S7 Fig Inconsistency (incoherence) assessments for CMT at the 12th week of triamcinolone acetonide treatment by different routes of administration. **Footnote:** IVTA: Intravitreal injection triamcinolone; RITA: Retrobulbar injections triamcinolone; STiTA: Sub-Tenon’s infusion of triamcinolone; PLA: Placebo; CMT: Central macular thickness.
(TIF)

S8 Fig Inconsistency (incoherence) assessments for CMT at the 24th week of triamcinolone acetonide treatment by different routes of administration. **Footnote:** IVTA: Intravitreal injection triamcinolone; RITA: Retrobulbar injections triamcinolone; PLA: Placebo; CMT: Central macular thickness.
(TIF)

S1 Table PRISMA checklist for this network meta-analysis. (DOCX)

S2 Table Search strategy. (DOCX)

S3 Table Risk of bias assessments. **Footnote:** D1: Risk of bias arising from the randomization process; D2: Risk of bias due to deviations from the intended interventions; D3: Risk of bias due to missing outcome data; D4: Risk of bias in measurement of the outcome; D5: Risk of bias in selection of the reported result; Overall: Overall risk of bias.
(DOCX)

S4 Table GRADE assessments for BCVA at the 12th week of triamcinolone acetonide treatment by different routes of administration. **Notes:** 1, Risk of bias; 2, Contributing direct evidence of moderate quality; 3, Imprecision.
(DOCX)

S5 Table GRADE assessments for BCVA at the 24th week of triamcinolone acetonide treatment by different routes of administration. **Notes:** 1, Risk of bias; 2, Contributing direct evidence of moderate quality; 3, Imprecision.
(DOCX)

S6 Table GRADE assessments for CMT at the 12th week of triamcinolone acetonide treatment by different routes of administration. **Notes:** 1, Risk of bias; 2, Contributing direct evidence of moderate quality; 3, Imprecision.
(DOCX)

S7 Table GRADE assessments for CMT at the 24th week of triamcinolone acetonide treatment by different routes of administration. **Notes:** 1, Risk of bias; 2, Contributing direct evidence of moderate quality; 3, Imprecision.
(DOCX)

S8 Table Bayesian methods SUCRA value for BCVA at the 12th week of triamcinolone acetonide treatment by different routes of administration. **Footnote:** BCVA: Best corrected visual acuity; IVTA: Intravitreal injection triamcinolone; OFTA: Orbital floor triamcinolone; RITA: Retrobulbar injections triamcinolone; SCTA: Suprachoroidal triamcinolone; STiTA: Sub-Tenon’s infusion of triamcinolone; PLA: Placebo.
(DOCX)

S9 Table Bayesian methods SUCRA value for BCVA at the 24th week of triamcinolone acetonide treatment by different routes of administration. **Footnote:** BCVA: Best corrected visual acuity; IVTA: Intravitreal injection triamcinolone; OFTA: Orbital floor triamcinolone; RITA: Retrobulbar injections triamcinolone; SCTA: Suprachoroidal triamcinolone; STiTA: Sub-Tenon’s infusion of triamcinolone; PLA: Placebo.
(DOCX)

S10 Table Bayesian methods SUCRA value for CMT at the 12th week of triamcinolone acetonide treatment by different routes of administration. **Footnote:** CMT: Central macular thickness; IVTA: Intravitreal injection triamcinolone; OFTA: Orbital floor triamcinolone; RITA: Retrobulbar injections triamcinolone; SCTA: Suprachoroidal triamcinolone; STiTA: Sub-Tenon’s infusion of triamcinolone; PLA: Placebo.
(DOCX)

S11 Table Bayesian methods SUCRA value for CMT at the 24th week of triamcinolone acetonide treatment by different routes of administration. **Footnote:** CMT: Central macular thickness; IVTA: Intravitreal injection triamcinolone; OFTA: Orbital floor triamcinolone; RITA: Retrobulbar injections triamcinolone; SCTA: Suprachoroidal triamcinolone; STiTA: Sub-Tenon’s infusion of triamcinolone; PLA: Placebo.
(DOCX)

S12 Table Exclusion of studies with non diabetic macular edema-Outcome: BCVA at the 12th week (Mean Difference; 95% confidence interval). **Footnote:** BCVA: Best corrected visual acuity; IVTA: Intravitreal injection triamcinolone; RITA: Retrobulbar injections triamcinolone; SCTA: Suprachoroidal triamcinolone; STiTA: Sub-Tenon’s infusion of triamcinolone; PLA: Placebo.
(DOCX)

S13 Table Exclusion of studies with non diabetic macular edema-Outcome: BCVA at the 24th week (Mean Difference; 95% confidence interval). **Footnote:** BCVA: Best corrected visual acuity; IVTA: Intravitreal injection triamcinolone; RITA: Retrobulbar injections triamcinolone; SCTA: Suprachoroidal triamcinolone; STiTA: Sub-Tenon’s infusion of triamcinolone; PLA: Placebo.
(DOCX)

S14 Table Exclusion of studies with non diabetic macular edema-Outcome: CMT at the 12th week (Mean Difference; 95% confidence interval). **Footnote:** CMT: Central macular thickness; IVTA: Intravitreal injection triamcinolone; RITA: Retrobulbar injections triamcinolone; SCTA: Suprachoroidal triamcinolone; STiTA: Sub-Tenon’s infusion of triamcinolone; PLA: Placebo.
(DOCX)

S15 Table Exclusion of studies with non diabetic macular edema-Outcome: CMT at the 24th week (Mean Difference; 95% confidence interval). **Footnote:** CMT: Central macular thickness; IVTA: Intravitreal injection triamcinolone; RITA: Retrobulbar injections triamcinolone; SCTA: Suprachoroidal triamcinolone; STiTA: Sub-Tenon’s infusion of triamcinolone; PLA: Placebo.
(DOCX)

S16 Table Exclusion of studies with fewer than 20 eyes-Outcome: CMT at the 12th week (Mean Difference; 95% confidence interval). **Footnote:** CMT: Central macular thickness; IVTA: Intravitreal injection triamcinolone; OFTA: Orbital floor triamcinolone; RITA: Retrobulbar injections triamcinolone; SCTA: Suprachoroidal triamcinolone; STiTA: Sub-Tenon’s infusion of triamcinolone; PLA: Placebo.
(DOCX)

S17 Table Exclusion of studies with fewer than 20 eyes-Outcome: CMT at the 24th week (Mean Difference; 95% confidence interval). **Footnote:** CMT: Central macular thickness; IVTA: Intravitreal injection triamcinolone; OFTA: Orbital floor triamcinolone; RITA: Retrobulbar injections triamcinolone; SCTA: Suprachoroidal triamcinolone; STiTA: Sub-Tenon’s infusion of triamcinolone; PLA: Placebo.
(DOCX)

S18 Table Exclusion of studies combined with laser therapy-Outcome: BCVA at the 12th week (Mean Difference; 95% confidence interval). **Footnote:** BCVA: Best corrected visual acuity; IVTA: Intravitreal injection triamcinolone; OFTA: Orbital floor triamcinolone; RITA: Retrobulbar injections triamcinolone; SCTA: Suprachoroidal triamcinolone; STiTA: Sub-Tenon’s infusion of triamcinolone.
(DOCX)

S19 Table Exclusion of studies combined with laser therapy-Outcome: BCVA at the 24th week (Mean Difference; 95% confidence interval). **Footnote:** BCVA: Best corrected visual acuity; IVTA: Intravitreal injection triamcinolone; OFTA: Orbital floor triamcinolone; RITA: Retrobulbar injections triamcinolone; SCTA: Suprachoroidal triamcinolone; STiTA: Sub-Tenon’s infusion of triamcinolone.
(DOCX)

S20 Table Exclusion of studies combined with laser therapy-Outcome: CMT at the 12th week (Mean Difference; 95% confidence interval). **Footnote:** CMT: Central macular thickness; IVTA: Intravitreal injection triamcinolone; OFTA: Orbital floor triamcinolone; RITA: Retrobulbar injections triamcinolone; SCTA: Suprachoroidal triamcinolone; STiTA: Sub-Tenon’s infusion of triamcinolone; PLA: Placebo.
(DOCX)

S21 Table Exclusion of studies combined with laser therapy-Outcome: CMT at the 24th week (Mean Difference; 95% confidence interval). **Footnote:** CMT: Central macular thickness; IVTA: Intravitreal injection triamcinolone; OFTA: Orbital floor triamcinolone; RITA: Retrobulbar injections triamcinolone; SCTA: Suprachoroidal triamcinolone; STiTA: Sub-Tenon’s infusion of triamcinolone.
(DOCX)

S22 Table Exclusion of studies with lost populations- Outcome: BCVA at the 12th week (Mean Difference; 95% confidence interval). **Footnote:** BCVA: Best corrected visual acuity; IVTA: Intravitreal injection triamcinolone; OFTA: Orbital floor triamcinolone; RITA: Retrobulbar injections triamcinolone; SCTA: Suprachoroidal triamcinolone; STiTA: Sub-Tenon’s infusion of triamcinolone; PLA: Placebo.
(DOCX)

S23 Table Exclusion of studies with lost populations- Outcome: BCVA at the 24th week (Mean Difference; 95% confidence interval). **Footnote:** BCVA: Best corrected visual acuity; IVTA: Intravitreal injection triamcinolone; OFTA: Orbital floor triamcinolone; RITA: Retrobulbar injections triamcinolone; SCTA: Suprachoroidal triamcinolone; STiTA: Sub-Tenon’s infusion of triamcinolone; PLA: Placebo.
(DOCX)

S24 Table Exclusion of studies with lost populations- Outcome: CMT at the 12th week (Mean Difference; 95% confidence interval). **Footnote:** CMT: Central macular thickness; IVTA: Intravitreal injection triamcinolone; OFTA: Orbital floor triamcinolone; RITA: Retrobulbar injections triamcinolone; SCTA: Suprachoroidal triamcinolone; STiTA: Sub-Tenon’s infusion of triamcinolone; PLA: Placebo.
(DOCX)

S25 Table Exclusion of studies with lost populations- Outcome: CMT at the 24th week (Mean Difference; 95% confidence interval). Footnote: CMT: Central macular thickness; IVTA: Intravitreal injection triamcinolone; OFTA: Orbital floor triamcinolone; RITA: Retrobulbar injections triamcinolone; SCTA: Suprachoroidal triamcinolone; STiTA: Sub-Tenon’s infusion of triamcinolone; PLA: Placebo.
(DOCX)
