# Supplementary figures and images for: Efficacy of different routes of triamcinolone acetonide administration on macular edema: A systematic review and network meta-analysis
Source: PLoS One. 2025 Jan 24;20(1):e0317782. doi: 10.1371/journal.pone.0317782 (PMC11760001; doi:10.1371/journal.pone.0317782)

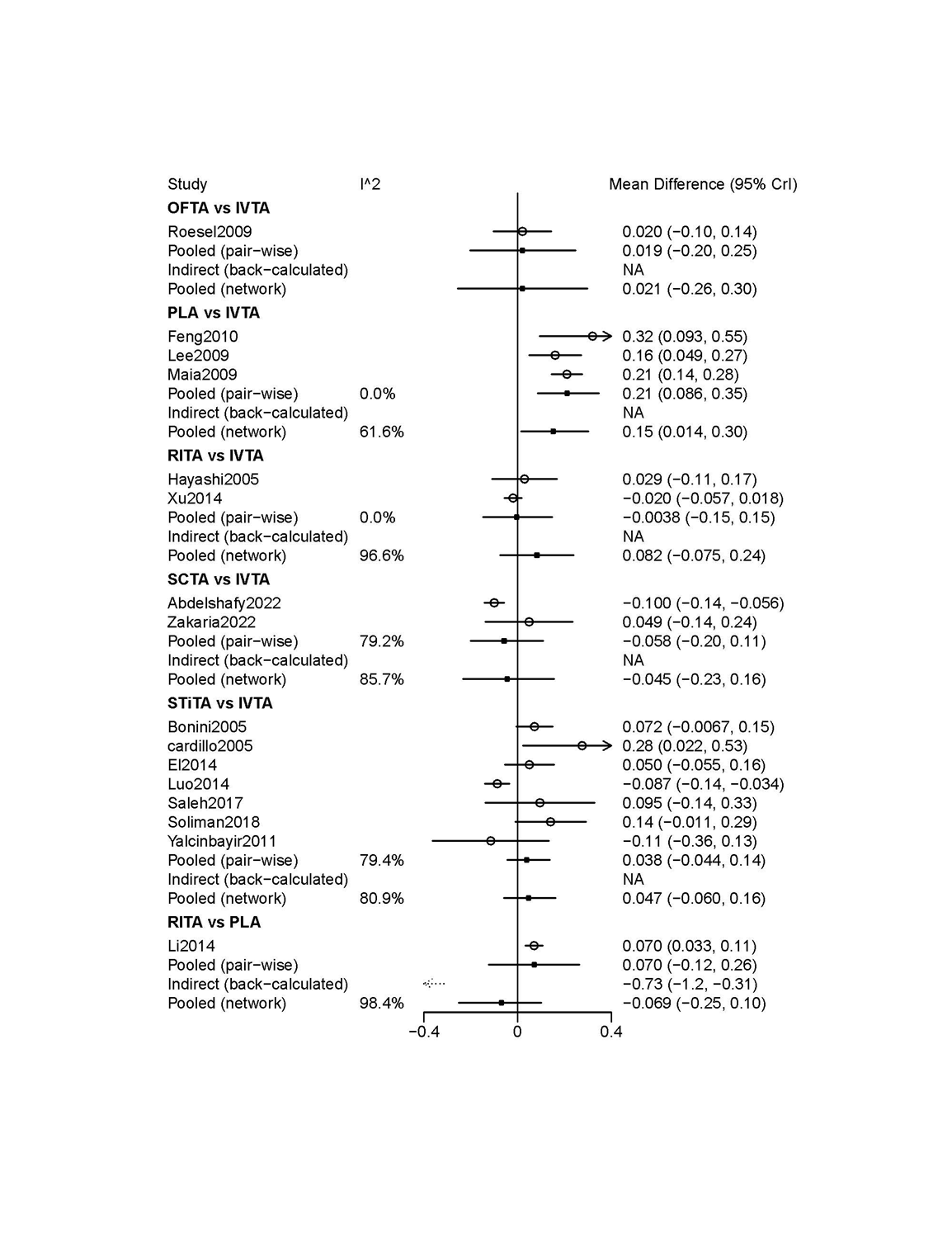

Supplement: S1 Fig — Footnote: OFTA: Orbital floor triamcinolone; IVTA: Intravitreal injection triamcinolone; RITA: Retrobulbar injections triamcinolone; SCTA: Suprachoroidal triamcinolone; STiTA: Sub-Tenon’s infusion of triamcinolone; PLA: Placebo; BCVA: Best corrected visual acuity. (TIF) [file pone.0317782.s001.tif]

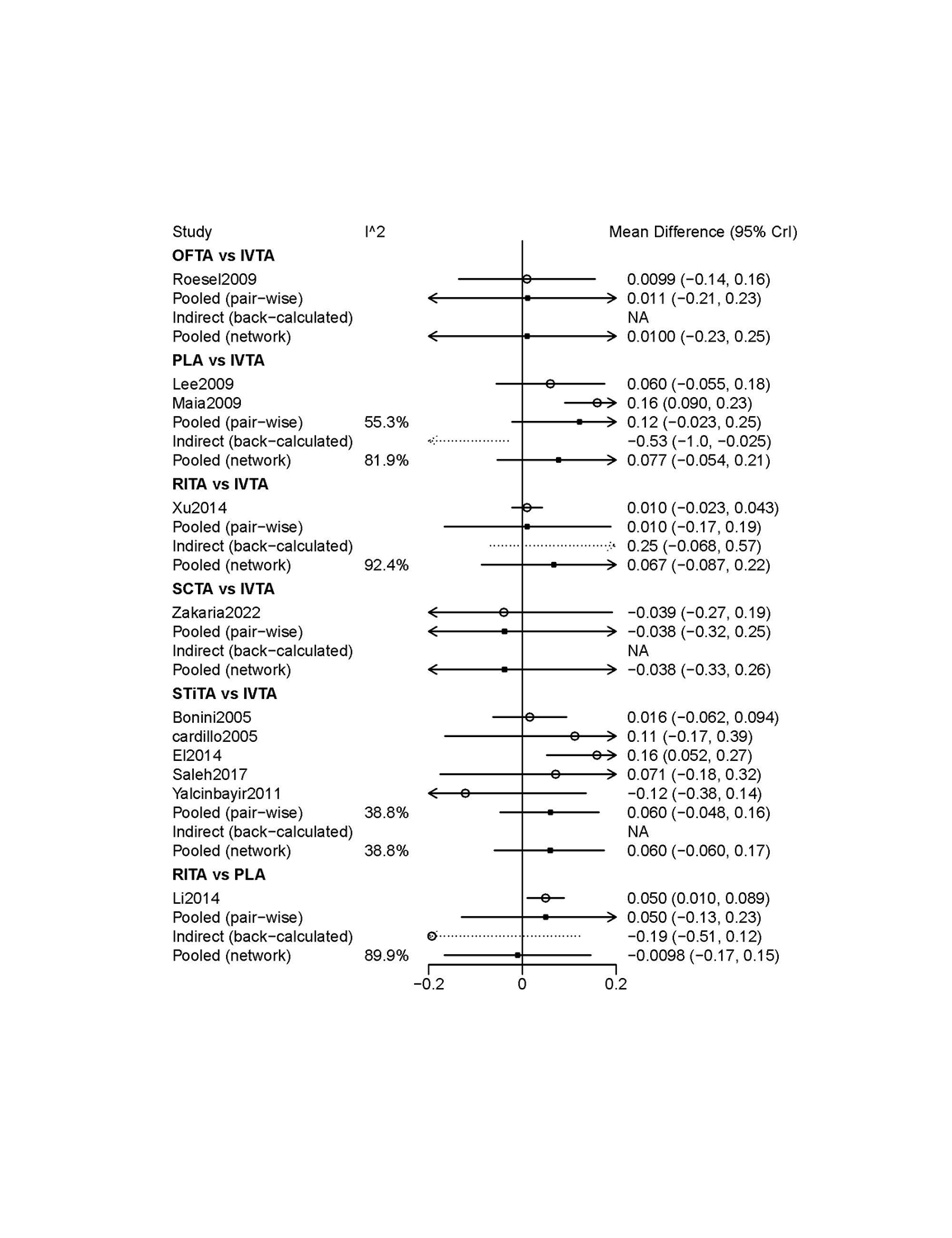

Supplement: S2 Fig — Footnote: OFTA: Orbital floor triamcinolone; IVTA: Intravitreal injection triamcinolone; RITA: Retrobulbar injections triamcinolone; SCTA: Suprachoroidal triamcinolone; STiTA: Sub-Tenon’s infusion of triamcinolone; PLA: Placebo; BCVA: Best corrected visual acuity. (TIF) [file pone.0317782.s002.tif]

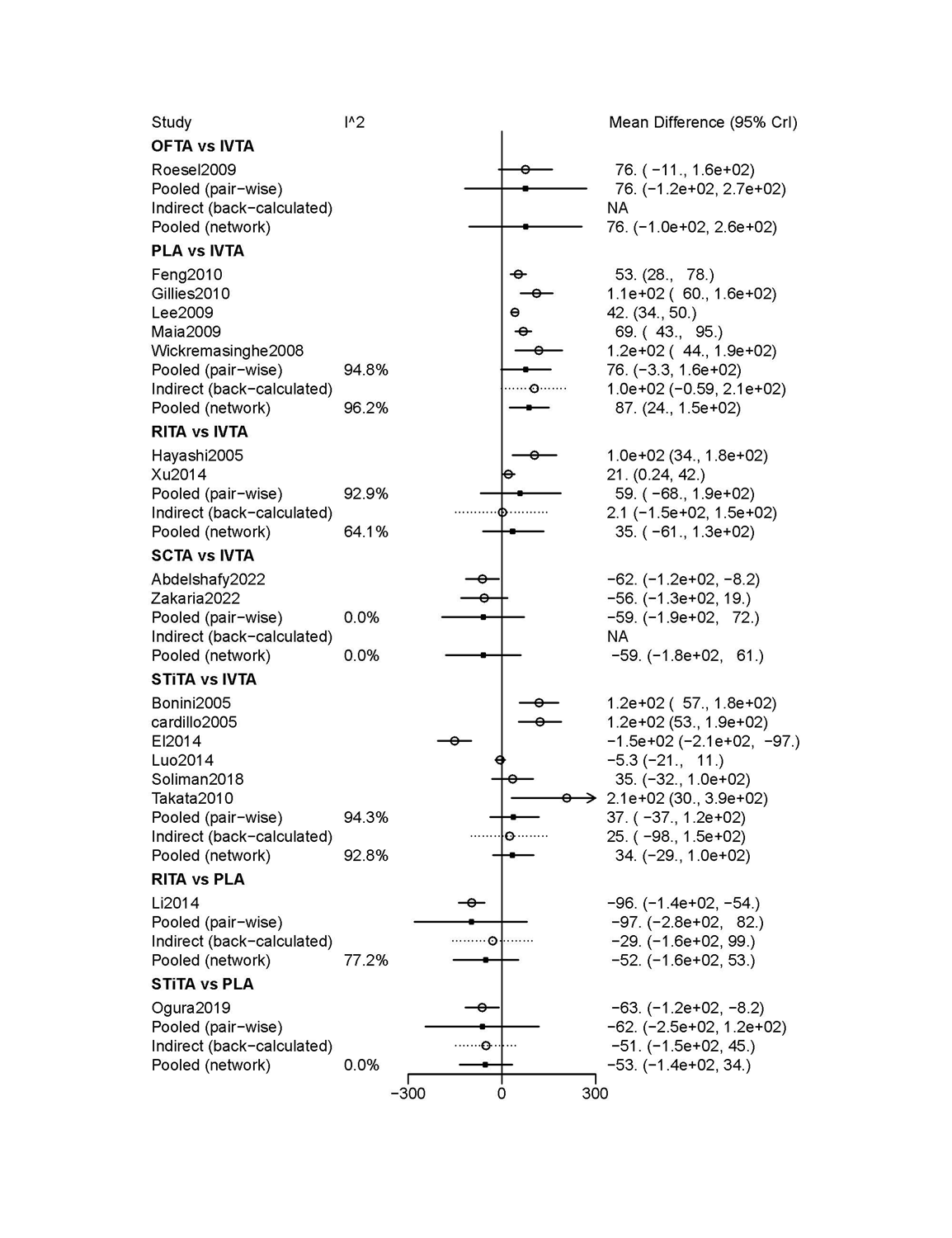

Supplement: S3 Fig — Footnote: OFTA: Orbital floor triamcinolone; IVTA: Intravitreal injection triamcinolone; RITA: Retrobulbar injections triamcinolone; SCTA: Suprachoroidal triamcinolone; STiTA: Sub-Tenon’s infusion of triamcinolone; PLA: Placebo; CMT: Central macular thickness. (TIF) [file pone.0317782.s003.tif]

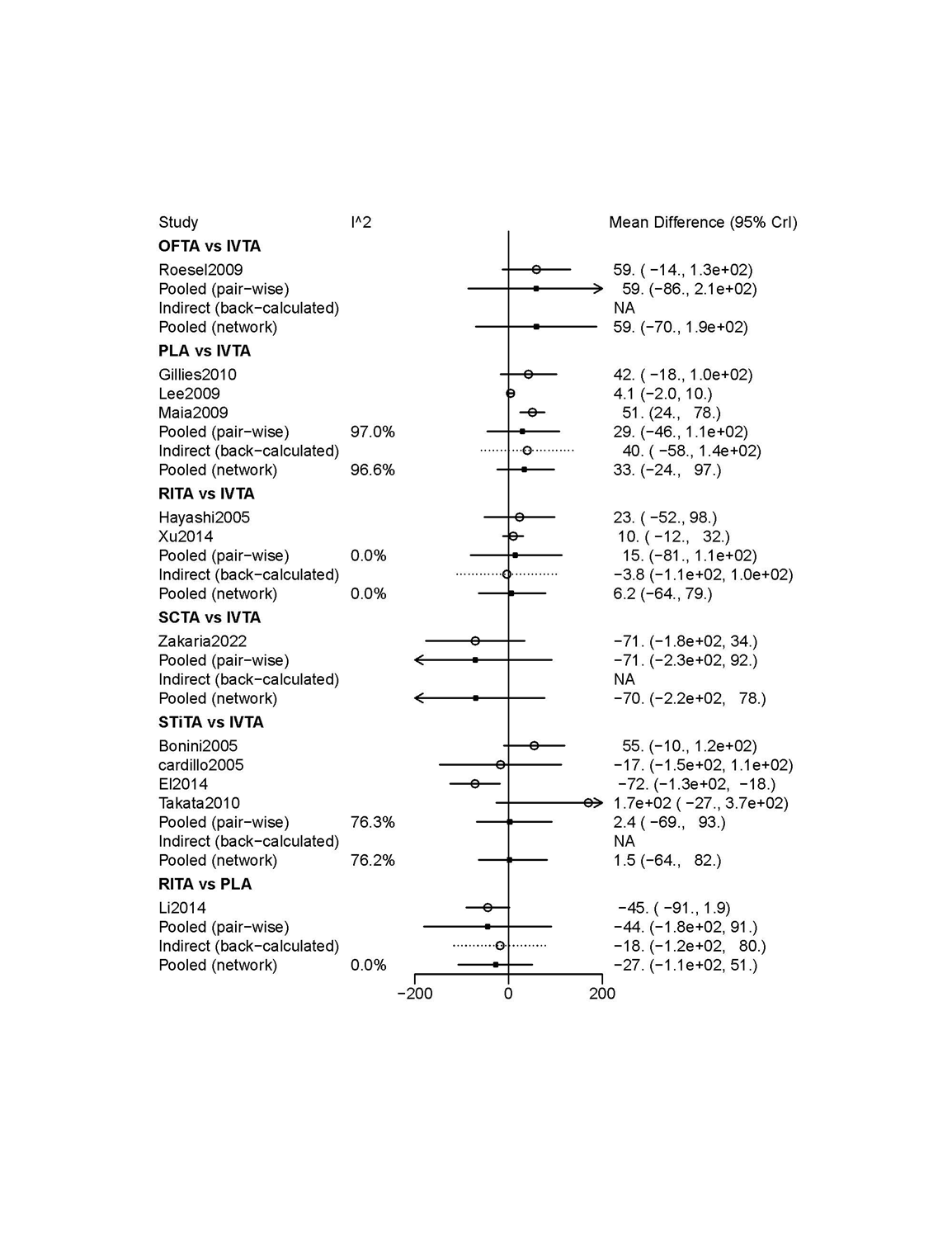

Supplement: S4 Fig — Footnote: OFTA: Orbital floor triamcinolone; IVTA: Intravitreal injection triamcinolone; RITA: Retrobulbar injections triamcinolone; SCTA: Suprachoroidal triamcinolone; STiTA: Sub-Tenon’s infusion of triamcinolone; PLA: Placebo; CMT: Central macular thickness. (TIF) [file pone.0317782.s004.tif]

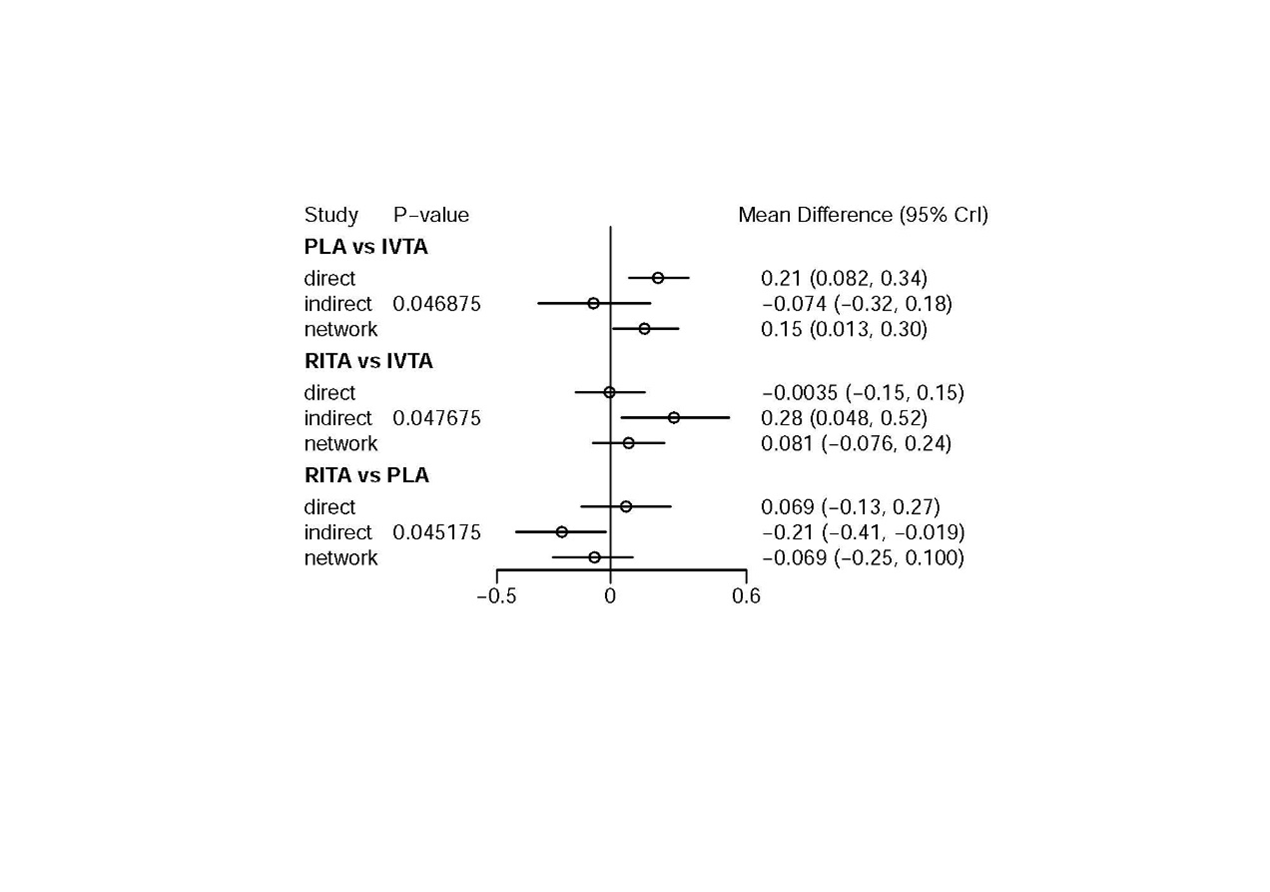

Supplement: S5 Fig — Footnote: IVTA: Intravitreal injection triamcinolone; RITA: Retrobulbar injections triamcinolone; PLA: Placebo; BCVA: Best corrected visual acuity. (TIF) [file pone.0317782.s005.tif]

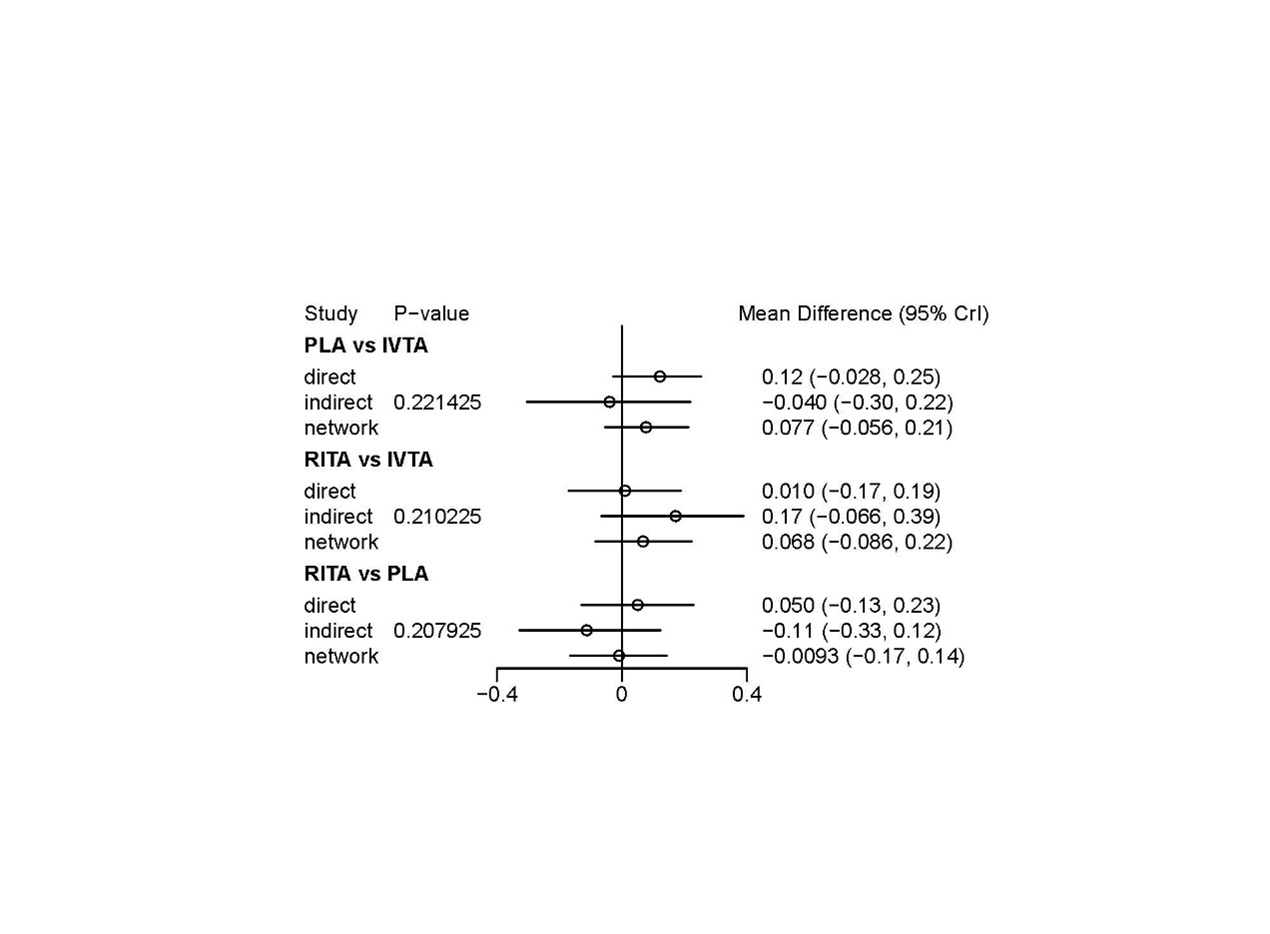

Supplement: S6 Fig — Footnote: IVTA: Intravitreal injection triamcinolone; RITA: Retrobulbar injections triamcinolone; PLA: Placebo; BCVA: Best corrected visual acuity. (TIF) [file pone.0317782.s006.tif]

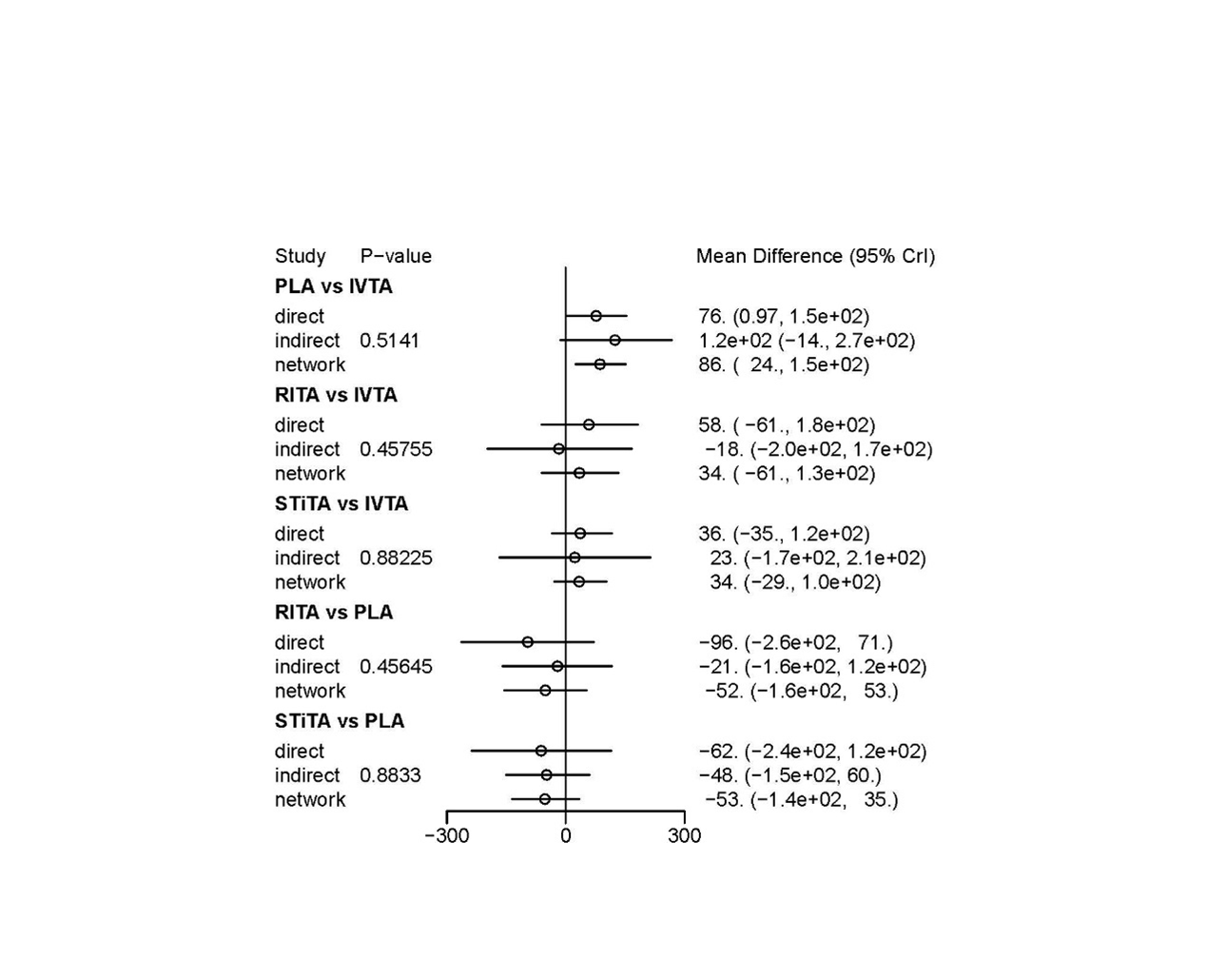

Supplement: S7 Fig — Footnote: IVTA: Intravitreal injection triamcinolone; RITA: Retrobulbar injections triamcinolone; STiTA: Sub-Tenon’s infusion of triamcinolone; PLA: Placebo; CMT: Central macular thickness. (TIF) [file pone.0317782.s007.tif]

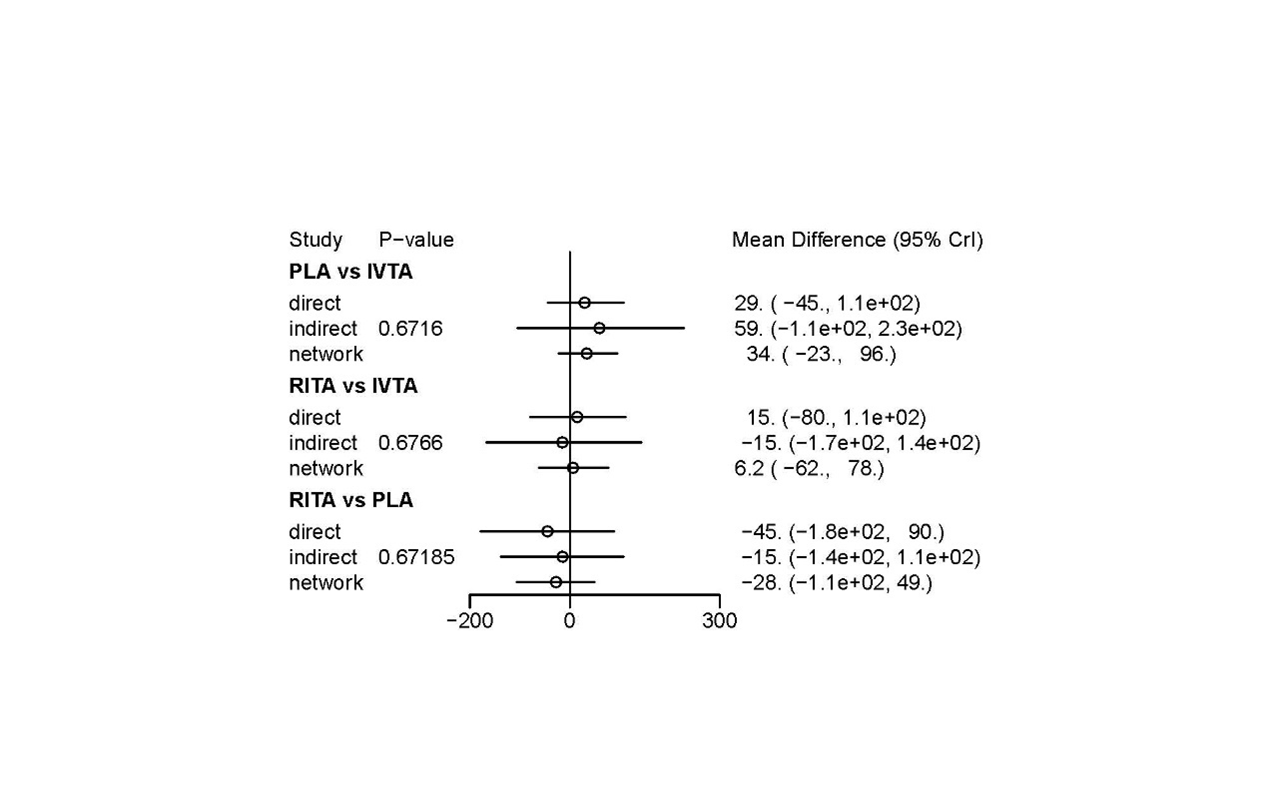

Supplement: S8 Fig — Footnote: IVTA: Intravitreal injection triamcinolone; RITA: Retrobulbar injections triamcinolone; PLA: Placebo; CMT: Central macular thickness. (TIF) [file pone.0317782.s008.tif]
