# Supplementary material for: Efficacy of different routes of triamcinolone acetonide administration on macular edema: A systematic review and network meta-analysis
Source: PLoS One. 2025 Jan 24;20(1):e0317782. doi: 10.1371/journal.pone.0317782 (PMC11760001; doi:10.1371/journal.pone.0317782)
Supplement: S2 Table — (DOCX) [file pone.0317782.s010.docx]

## Supplementary Table 2. Search strategy

| Database | Search strategy |
| --- | --- |
| PubMed | ("macular edema"[Text Word] OR "macular edema"[MeSH Major Topic] OR ("Cystoid Macular Edema"[Text Word] OR "Irvine*Gass Syndrome"[Text Word])) AND ("triamcinolone acetonide"[Text Word] OR "triamcinolone acetonide"[MeSH Major Topic] OR "Tricort*40"[Text Word]) AND ("randomized controlled trial"[Publication Type] OR "controlled clinical trial"[Publication Type] OR "clinical trials as topic"[MeSH Terms] OR "randomized"[Title/Abstract] OR "randomised"[Title/Abstract] OR "placebo"[Title/Abstract] OR "randomly"[Title/Abstract] OR "trial"[Title] OR "drug therapy"[MeSH Subheading] OR "groups"[Title/Abstract]) |
| Embase (Ovid SP) | (exp macular edema/ or macular edema.mp. or Cystoid Macular Edema.mp. or Irvine*Gass Syndrome.mp.) AND (exp triamcinolone acetonide/ or triamcinolone acetonide.mp. or Tricort*40.mp.) AND (exp Clinical trial/ or Randomized controlled trial/ or Randomization/ or Randomi?ed controlled trial$.tw. or Rct.tw. or Random allocation.tw. or Randomly allocated.tw. or Allocated randomly.tw. or (allocated adj2 random).tw.) |
| Medline (Ovid SP) | (exp macular edema/ or macular edema.mp. or Cystoid Macular Edema.mp. or Irvine*Gass Syndrome.mp.) AND (exp triamcinolone acetonide/ or triamcinolone acetonide.mp. or Tricort*40.mp.) AND (exp Clinical trial/ or Randomized controlled trial/ or Randomization/ or Randomi?ed controlled trial$.tw. or Rct.tw. or Random allocation.tw. or Randomly allocated.tw. or Allocated randomly.tw. or (allocated adj2 random).tw.) |
| Cochrane Central Register of Controlled Trials (via OVID) | (exp macular edema/ or macular edema.mp. or Cystoid Macular Edema.mp. or Irvine*Gass Syndrome.mp.) AND (exp triamcinolone acetonide/ or triamcinolone acetonide.mp. or Tricort*40.mp.) AND (exp Clinical trial/ or Randomized controlled trial/ or Randomization/ or Randomi?ed controlled trial$.tw. or Rct.tw. or Random allocation.tw. or Randomly allocated.tw. or Allocated randomly.tw. or (allocated adj2 random).tw.) |
