# Supplementary material for: Efficacy of different routes of triamcinolone acetonide administration on macular edema: A systematic review and network meta-analysis
Source: PLoS One. 2025 Jan 24;20(1):e0317782. doi: 10.1371/journal.pone.0317782 (PMC11760001; doi:10.1371/journal.pone.0317782)
Supplement: S3 Table — Footnote: D1: Risk of bias arising from the randomization process; D2: Risk of bias due to deviations from the intended interventions; D3: Risk of bias due to missing outcome data; D4: Risk of bias in measurement of the outcome; D5: Risk of bias in selection of the reported result; Overall: Overall risk of bias. (DOCX) [file pone.0317782.s011.docx]

## Supplementary Table 3. Risk of bias assessments

| **Study ID** | **D1** | **D2** | **D3** | **D4** | **D5** | **Overall** |
| --- | --- | --- | --- | --- | --- | --- |
| Abdelshafy 2022 | Some concerns | Some concerns | Low | Low | Low | Some concerns |
| Bonini 2005 | Low | Some concerns | Low | Low | Low | Some concerns |
| Cardillo 2005 | Low | Low | Low | Low | Low | Low |
| EI 2014 | Some concerns | Some concerns | Low | Low | Low | Some concerns |
| Feng 2010 | Some concerns | Some concerns | Low | Low | Some concerns | Some concerns |
| Gillies 2010 | Low | Low | Low | Low | Low | Low |
| Hayashi 2005 | Low | Low | Low | Low | Low | Low |
| Lee 2009 | Some concerns | Some concerns | Low | Low | Low | Some concerns |
| Li 2014 | Some concerns | Some concerns | Low | Low | Low | Some concerns |
| Luo 2014 | Some concerns | Low | Low | Low | Low | Some concerns |
| Maia 2009 | Low | Low | Low | Low | Low | Low |
| Ogura 2019 | Some concerns | Low | Low | Low | Low | Some concerns |
| Roesel 2009 | Some concerns | Some concerns | Low | Some concerns | Low | Some concerns |
| Saleh 2017 | Some concerns | Some concerns | Low | Low | Low | Some concerns |
| Soliman 2018 | Some concerns | Some concerns | Low | Low | Low | Some concerns |
| Takata 2010 | Some concerns | Some concerns | Low | Low | Low | Some concerns |
| Wickremasinghe 2008 | Some concerns | Some concerns | Low | Low | Low | Some concerns |
| Xu 2014 | Some concerns | Some concerns | Low | Low | Low | Some concerns |
| Yalcinbayir 2011 | Some concerns | Some concerns | Low | Low | Some concerns | Some concerns |
| Zakaria 2022 | Some concerns | Some concerns | Low | Low | Low | Some concerns |

**Footnote:** D1: Risk of bias arising from the randomization process; D2: Risk of bias due to deviations from the intended interventions; D3: Risk of bias due to missing outcome data; D4: Risk of bias in measurement of the outcome; D5: Risk of bias in selection of the reported result; Overall: Overall risk of bias.
