# Supplementary material for: Efficacy of different routes of triamcinolone acetonide administration on macular edema: A systematic review and network meta-analysis
Source: PLoS One. 2025 Jan 24;20(1):e0317782. doi: 10.1371/journal.pone.0317782 (PMC11760001; doi:10.1371/journal.pone.0317782)
Supplement: S4 Table — Notes: 1, Risk of bias; 2, Contributing direct evidence of moderate quality; 3, Imprecision. (DOCX) [file pone.0317782.s012.docx]

## Supplementary Table 4. GRADE assessments for BCVA at the 12th week of triamcinolone acetonide treatment by different routes of administration

| **Comparison** | **Direct estimate** | **Certainty** | **Indirect estimate** | **Certainty** | **Network estimate** | **Certainty** |
| --- | --- | --- | --- | --- | --- | --- |
| IVTA vs OFTA | -0.02 (-0.29, 0.26) | Moderate^1^ | - | - | -0.02 (-0.29, 0.26) | Low^3^ |
| IVTA vs PLA | -0.21 (-0.34, -0.08) | Moderate^1^ | 0.07 (-0.18, 0.32) | Moderate^2^ | **-0.15 (-0.30,-0.01 )** | Moderate |
| IVTA vs RITA | 0.00 (-0.15, 0.15) | Moderate^1^ | -0.28 (-0.52, -0.05) | Moderate^2^ | -0.08 (-0.24,0.08 ) | Low^3^ |
| IVTA vs SCTA | 0.05 (-0.16, 0.24) | Moderate^1^ | - | - | 0.05 (-0.16, 0.24) | Low^3^ |
| IVTA vs STiTA | -0.05 (-0.16, 0.06) | Moderate^1^ | - | - | -0.05 (-0.16, 0.06) | Low^3^ |
| RITA vs PLA | 0.07 (-0.13, 0.27) | Moderate^1^ | -0.21 (-0.41, -0.02) | Moderate^2^ | -0.07 (-0.25, 0.10) | Low^3^ |
| OFTA vs PLA | - | - | -0.13 (-0.45, 0.17) | Moderate^2^ | -0.13 (-0.45, 0.17) | Low^3^ |
| OFTA vs RITA | - | - | -0.06 (-0.38, 0.26) | Moderate^2^ | -0.06 (-0.38, 0.26) | Low^3^ |
| OFTA vs SCTA | - | - | 0.07 (-0.28, 0.40) | Moderate^2^ | 0.07 (-0.28, 0.40) | Low^3^ |
| OFTA vs STiTA | - | - | -0.03 (-0.33, 0.27) | Moderate^2^ | -0.03 (-0.33, 0.27) | Low^3^ |
| PLA vs SCTA | - | - | 0.20 (-0.04, 0.44) | Moderate^2^ | 0.20 (-0.04, 0.44) | Low^3^ |
| PLA vs STiTA | - | - | 0.10 (-0.08, 0.29) | Moderate^2^ | 0.10 (-0.08, 0.29) | Low^3^ |
| RITA vs SCTA | - | - | 0.13 (-0.13, 0.37) | Moderate^2^ | 0.13 (-0.13, 0.37) | Low^3^ |
| RITA vs STiTA | - | - | 0.03 (-0.16, 0.22) | Moderate^2^ | 0.03 (-0.16, 0.22) | Low^3^ |
| SCTA vs STiTA | - | - | -0.09 (-0.32, 0.13) | Moderate^2^ | -0.09 (-0.32, 0.13) | Low^3^ |

**Notes:** 1, Risk of bias; 2, Contributing direct evidence of moderate quality; 3, Imprecision.
