# Supplementary material for: Efficacy of different routes of triamcinolone acetonide administration on macular edema: A systematic review and network meta-analysis
Source: PLoS One. 2025 Jan 24;20(1):e0317782. doi: 10.1371/journal.pone.0317782 (PMC11760001; doi:10.1371/journal.pone.0317782)
Supplement: S5 Table — Notes: 1, Risk of bias; 2, Contributing direct evidence of moderate quality; 3, Imprecision. (DOCX) [file pone.0317782.s013.docx]

**Supplementary Table 5. GRADE assessments for** **BCVA at the 24th week of triamcinolone acetonide treatment by different routes of administration**

| **Comparison** | **Direct estimate** | **Certainty** | **Indirect estimate** | **Certainty** | **Network estimate** | **Certainty** |
| --- | --- | --- | --- | --- | --- | --- |
| IVTA vs OFTA | -0.01 (-0.25, 0.23) | Moderate^1^ | - | - | -0.01 (-0.25, 0.23) | Low^3^ |
| IVTA vs PLA | -0.12 (-0.25,0.028 ) | Moderate^1^ | 0.04 (-0.22, 0.30) | Moderate^2^ | -0.08 (-0.21, 0.05) | Low^3^ |
| IVTA vs RITA | -0.01(-0.19, 0.17) | Moderate^1^ | -0.17 (-0.39, 0.07) | Moderate^2^ | -0.07 (-0.23, 0.09) | Low^3^ |
| IVTA vs SCTA | 0.04 (-0.26, 0.34) | Moderate^1^ | - | - | 0.04 (-0.26, 0.34) | Low^3^ |
| IVTA vs STiTA | -0.06 (-0.17, 0.06) | Moderate^1^ | - | - | -0.06 (-0.17, 0.06) | Low^3^ |
| RITA vs PLA | 0.05(-0.13, 0.23) | Moderate^1^ | -0.11 (-0.33, 0.12) | Moderate^2^ | -0.01 (-0.17, 0.15) | Low^3^ |
| OFTA vs PLA | - | - | -0.07 (-0.35, 0.21) | Moderate^2^ | -0.07 (-0.35, 0.21) | Low^3^ |
| OFTA vs RITA | - | - | -0.06 (-0.35, 0.23) | Moderate^2^ | -0.06 (-0.35, 0.23) | Low^3^ |
| OFTA vs SCTA | - | - | 0.05 (-0.33, 0.43) | Moderate^2^ | 0.05 (-0.33, 0.43) | Low^3^ |
| OFTA vs STiTA | - | - | -0.05 (-0.31, 0.22) | Moderate^2^ | -0.05 (-0.31, 0.22) | Low^3^ |
| PLA vs SCTA | - | - | 0.12 (-0.21, 0.45) | Moderate^2^ | 0.12 (-0.21, 0.45) | Low^3^ |
| PLA vs STiTA | - | - | 0.02 (-0.15, 0.2) | Moderate^2^ | 0.02 (-0.15, 0.2) | Low^3^ |
| RITA vs SCTA | - | - | 0.11 (-0.23, 0.44) | Moderate^2^ | 0.11 (-0.23, 0.44) | Low^3^ |
| RITA vs STiTA | - | - | 0.01 (-0.18, 0.21) | Moderate^2^ | 0.01 (-0.18, 0.21) | Low^3^ |
| SCTA vs STiTA | - | - | -0.1 (-0.42, 0.22) | Moderate^2^ | -0.1 (-0.42, 0.22) | Low^3^ |

**Notes:** 1, Risk of bias; 2, Contributing direct evidence of moderate quality; 3, Imprecision.
