# Supplementary material for: Efficacy of different routes of triamcinolone acetonide administration on macular edema: A systematic review and network meta-analysis
Source: PLoS One. 2025 Jan 24;20(1):e0317782. doi: 10.1371/journal.pone.0317782 (PMC11760001; doi:10.1371/journal.pone.0317782)
Supplement: S6 Table — Notes: 1, Risk of bias; 2, Contributing direct evidence of moderate quality; 3, Imprecision. (DOCX) [file pone.0317782.s014.docx]

**Supplementary Table 6. GRADE assessments for CMT at the 12th week of triamcinolone acetonide treatment by different routes of administration**

| **Comparison** | **Direct estimate** | **Certainty** | **Indirect estimate** | **Certainty** | **Network estimate** | **Certainty** |
| --- | --- | --- | --- | --- | --- | --- |
| IVTA vs OFTA | -75.65 (-254.44, 103.12) | Moderate^1^ | - | - | -75.65 (-254.44, 103.12) | Low^3^ |
| IVTA vs PLA | -76. (-1.5e+02, -0.97) | Moderate^1^ | -1.2e+02 (-2.7e+02,14.) | Moderate^2^ | **-86.54 (-152.18, -23.82)** | Moderate |
| IVTA vs RITA | -58. (-1.8e+02, 61.) | Moderate^1^ | 18. (-1.7e+02, 2.0e+02) | Moderate^2^ | -34.66 (-133.75, 60.83) | Low^3^ |
| IVTA vs SCTA | 58.92 (-62.19, 178.78) | Moderate^1^ | - | - | 58.92 (-62.19, 178.78) | Low^3^ |
| IVTA vs STiTA | -36. (-1.2e+02, 35.) | Moderate^1^ | -23. (-2.1e+02,1.7e+02 ) | Moderate^2^ | -33.74 (-103.53, 29.61) | Low^3^ |
| STiTA vs PLA | -62. (-2.4e+02, 1.2e+02) | Moderate^1^ | -48. (-1.5e+02, 60.) | Moderate^2^ | -52.88 (-135.8, 34.19) | Low^3^ |
| RITA vs PLA | -96. (-2.6e+02, 71.) | Moderate^1^ | -21. (-1.6e+02, 1.2e+02) | Moderate^2^ | -51.76 (-155.56, 52.6) | Low^3^ |
| OFTA vs PLA | - | - | -11.03 (-201.95, 177.36) | Moderate^2^ | -11.03 (-201.95, 177.36) | Low^3^ |
| OFTA vs RITA | - | - | 41.02 (-164.53, 242.81) | Moderate^2^ | 41.02 (-164.53, 242.81) | Low^3^ |
| OFTA vs SCTA | - | - | 134.49 (-80.22, 350.42) | Moderate^2^ | 134.49 (-80.22, 350.42) | Low^3^ |
| OFTA vs STiTA | - | - | 41.98 (-151.74, 229.12) | Moderate^2^ | 41.98 (-151.74, 229.12) | Low^3^ |
| PLA vs SCTA | - | - | 145.5 (10.15, 283.04) | Moderate^2^ | **145.5 (10.15, 283.04)** | Moderate |
| RITA vs SCTA | - | - | 93.78 (-59.97, 249.95) | Moderate^2^ | 93.78 (-59.97, 249.95) | Low^3^ |
| RITA vs STiTA | - | - | 1.03 (-115.89, 115.24) | Moderate^2^ | 1.03 (-115.89, 115.24) | Low^3^ |
| SCTA vs STiTA | - | - | -92.55 (-233.08, 41.64) | Moderate^2^ | -92.55 (-233.08, 41.64) | Low^3^ |

**Notes:** 1, Risk of bias; 2, Contributing direct evidence of moderate quality; 3, Imprecision.
