# Supplementary material for: Efficacy of different routes of triamcinolone acetonide administration on macular edema: A systematic review and network meta-analysis
Source: PLoS One. 2025 Jan 24;20(1):e0317782. doi: 10.1371/journal.pone.0317782 (PMC11760001; doi:10.1371/journal.pone.0317782)
Supplement: S7 Table — Notes: 1, Risk of bias; 2, Contributing direct evidence of moderate quality; 3, Imprecision. (DOCX) [file pone.0317782.s015.docx]

**Supplementary Table 7. GRADE assessments for CMT at the 24th week of triamcinolone acetonide treatment by different routes of administration**

| **Comparison** | **Direct estimate** | **Certainty** | **Indirect estimate** | **Certainty** | **Network estimate** | **Certainty** |
| --- | --- | --- | --- | --- | --- | --- |
| IVTA vs OFTA | -59.12 (-187.38, 69.1) | Moderate^1^ | - |  | -59.12 (-187.38, 69.1) | Low^3^ |
| IVTA vs PLA | -29. ( −1.1e+02, 45.) | Moderate^1^ | -59. (−2.3e+02, 1.1e+02) | Moderate^2^ | -33.38 (-95.41, 23.31) | Low^3^ |
| IVTA vs RITA | -15. (−1.1e+02, 80.) | Moderate^1^ | 15. (−1.4e+02, 1.7e+02) | Moderate^2^ | -6.09 (-78.27, 63.08) | Low^3^ |
| IVTA vs SCTA | 71.72 (-77.91, 219.49) | Moderate^1^ | - |  | 71.72 (-77.91, 219.49) | Low^3^ |
| IVTA vs STiTA | -1.01 (-81.17, 63.87) | Moderate^1^ | - | Moderate^2^ | -1.01 (-81.17, 63.87) | Low^3^ |
| RITA vs PLA | −45. (−1.8e+02, 90.) | Moderate^1^ | −15. (−1.4e+02, 1.1e+02) | Moderate^2^ | -27.3 (-106.98, 49.64) | Low^3^ |
| OFTA vs PLA | - |  | 25.43 (-118.37, 164.61) | Moderate^2^ | 25.43 (-118.37, 164.61) | Low^3^ |
| OFTA vs RITA | - |  | 52.82 (-94.64, 197.95) | Moderate^2^ | 52.82 (-94.64, 197.95) | Low^3^ |
| OFTA vs SCTA | - |  | 130.46 (-65.84, 326.61) | Moderate^2^ | 130.46 (-65.84, 326.61) | Low^3^ |
| OFTA vs STiTA | - |  | 57.98 (-96.66, 197.51) | Moderate^2^ | 57.98 (-96.66, 197.51) | Low^3^ |
| PLA vs SCTA | - |  | 105.71 (-53.58, 266.13) | Moderate^2^ | 105.71 (-53.58, 266.13) | Low^3^ |
| PLA vs STiTA | - |  | 33.06 (-66.77, 119.57) | Moderate^2^ | 33.06 (-66.77, 119.57) | Low^3^ |
| RITA vs SCTA | - |  | 77.9 (-85.78, 242.12) | Moderate^2^ | 77.9 (-85.78, 242.12) | Low^3^ |
| RITA vs STiTA | - |  | 5.64 (-102.59, 97.66) | Moderate^2^ | 5.64 (-102.59, 97.66) | Low^3^ |
| SCTA vs STiTA | - |  | -73.05 (-243.47, 87.15) | Moderate^2^ | -73.05 (-243.47, 87.15) | Low^3^ |

**Notes:** 1, Risk of bias; 2, Contributing direct evidence of moderate quality; 3, Imprecision.
