# Supplementary material for: Efficacy of different routes of triamcinolone acetonide administration on macular edema: A systematic review and network meta-analysis
Source: PLoS One. 2025 Jan 24;20(1):e0317782. doi: 10.1371/journal.pone.0317782 (PMC11760001; doi:10.1371/journal.pone.0317782)
Supplement: S8 Table — Footnote: BCVA: Best corrected visual acuity; IVTA: Intravitreal injection triamcinolone; OFTA: Orbital floor triamcinolone; RITA: Retrobulbar injections triamcinolone; SCTA: Suprachoroidal triamcinolone; STiTA: Sub-Tenon’s infusion of triamcinolone; PLA: Placebo. (DOCX) [file pone.0317782.s016.docx]

## Supplementary Table 8. Bayesian methods SUCRA value for BCVA at the 12th week of triamcinolone acetonide treatment by different routes of administration

| **The BCVA at 12th week (Mean Difference; 95% confidence interval)** | | |
| --- | --- | --- |
| **Intervention** | **Intervention vs PLA** | **SUCRA value** |
| IVTA | −0.15 (−0.30, −0.013) | 0.7067 |
| OFTA | −0.13 (−0.45, 0.17) | 0.5668 |
| RITA | −0.069 (−0.25, 0.10) | 0.3534 |
| SCTA | −0.20 (−0.44, 0.044) | 0.7970 |
| STiTA | −0.10 (−0.29, 0.076) | 0.4656 |
| PLA | - | 0.1105 |

**Footnote:** BCVA: Best corrected visual acuity; IVTA: Intravitreal injection triamcinolone; OFTA: Orbital floor triamcinolone; RITA: Retrobulbar injections triamcinolone; SCTA: Suprachoroidal triamcinolone; STiTA: Sub-Tenon’s infusion of triamcinolone; PLA: Placebo.
