# Supplementary material for: Efficacy of different routes of triamcinolone acetonide administration on macular edema: A systematic review and network meta-analysis
Source: PLoS One. 2025 Jan 24;20(1):e0317782. doi: 10.1371/journal.pone.0317782 (PMC11760001; doi:10.1371/journal.pone.0317782)
Supplement: S9 Table — Footnote: BCVA: Best corrected visual acuity; IVTA: Intravitreal injection triamcinolone; OFTA: Orbital floor triamcinolone; RITA: Retrobulbar injections triamcinolone; SCTA: Suprachoroidal triamcinolone; STiTA: Sub-Tenon’s infusion of triamcinolone; PLA: Placebo. (DOCX) [file pone.0317782.s017.docx]

## Supplementary Table 9. Bayesian methods SUCRA value for BCVA at the 24th week of triamcinolone acetonide treatment by different routes of administration

| **The BCVA at 24th week (Mean Difference; 95% confidence interval)** | | |
| --- | --- | --- |
| **Intervention** | **Intervention vs PLA** | **SUCRA value** |
| IVTA | −0.077 (−0.21, 0.054) | 0.7042 |
| OFTA | −0.068 (−0.35, 0.21) | 0.5757 |
| RITA | −0.0096 (−0.17, 0.15) | 0.3535 |
| SCTA | −0.12 (−0.45, 0.21) | 0.6895 |
| STiTA | −0.018 (−0.20, 0.15) | 0.3776 |
| PLA | - | 0.2994 |

**Footnote:** BCVA: Best corrected visual acuity; IVTA: Intravitreal injection triamcinolone; OFTA: Orbital floor triamcinolone; RITA: Retrobulbar injections triamcinolone; SCTA: Suprachoroidal triamcinolone; STiTA: Sub-Tenon’s infusion of triamcinolone; PLA: Placebo.
