# Supplementary material for: Efficacy of different routes of triamcinolone acetonide administration on macular edema: A systematic review and network meta-analysis
Source: PLoS One. 2025 Jan 24;20(1):e0317782. doi: 10.1371/journal.pone.0317782 (PMC11760001; doi:10.1371/journal.pone.0317782)
Supplement: S10 Table — Footnote: CMT: Central macular thickness; IVTA: Intravitreal injection triamcinolone; OFTA: Orbital floor triamcinolone; RITA: Retrobulbar injections triamcinolone; SCTA: Suprachoroidal triamcinolone; STiTA: Sub-Tenon’s infusion of triamcinolone; PLA: Placebo. (DOCX) [file pone.0317782.s018.docx]

## Supplementary Table 10. Bayesian methods SUCRA value for CMT at the 12th week of triamcinolone acetonide treatment by different routes of administration

| **The CMT at 12th week (Mean Difference; 95% confidence interval)** | | |
| --- | --- | --- |
| **Intervention** | **Intervention vs PLA** | **SUCRA value** |
| IVTA | -86.54 (-152.18, -23.82) | 0.7193 |
| OFTA | -11.03 (-201.95, 177.36) | 0.2998 |
| RITA | -51.76 (-155.56, 52.6) | 0.4668 |
| SCTA | -145.5 (-283.04, -10.15) | 0.9093 |
| STiTA | -52.88 (-135.8, 34.19) | 0.4600 |
| PLA | - | 0.1448 |

**Footnote:** CMT: Central macular thickness; IVTA: Intravitreal injection triamcinolone; OFTA: Orbital floor triamcinolone; RITA: Retrobulbar injections triamcinolone; SCTA: Suprachoroidal triamcinolone; STiTA: Sub-Tenon’s infusion of triamcinolone; PLA: Placebo.
