# Supplementary material for: Efficacy of different routes of triamcinolone acetonide administration on macular edema: A systematic review and network meta-analysis
Source: PLoS One. 2025 Jan 24;20(1):e0317782. doi: 10.1371/journal.pone.0317782 (PMC11760001; doi:10.1371/journal.pone.0317782)
Supplement: S11 Table — Footnote: CMT: Central macular thickness; IVTA: Intravitreal injection triamcinolone; OFTA: Orbital floor triamcinolone; RITA: Retrobulbar injections triamcinolone; SCTA: Suprachoroidal triamcinolone; STiTA: Sub-Tenon’s infusion of triamcinolone; PLA: Placebo. (DOCX) [file pone.0317782.s019.docx]

## Supplementary Table 11. Bayesian methods SUCRA value for CMT at the 24th week of triamcinolone acetonide treatment by different routes of administration

| **The CMT at 24th week (Mean Difference; 95% confidence interval)** | | |
| --- | --- | --- |
| **Intervention** | **Intervention vs PLA** | **SUCRA value** |
| IVTA | -33.38 (-95.41, 23.31) | 0.6030 |
| OFTA | 25.43 (-118.37, 164.61) | 0.1977 |
| RITA | -27.3 (-106.98, 49.64) | 0.5230 |
| SCTA | -105.71 (-266.13, 53.58) | 0.8685 |
| STiTA | -33.06 (-119.57, 66.77) | 0.5605 |
| PLA | - | 0.2473 |

**Footnote:** CMT: Central macular thickness; IVTA: Intravitreal injection triamcinolone; OFTA: Orbital floor triamcinolone; RITA: Retrobulbar injections triamcinolone; SCTA: Suprachoroidal triamcinolone; STiTA: Sub-Tenon’s infusion of triamcinolone; PLA: Placebo.
