# Supplementary material for: Efficacy of different routes of triamcinolone acetonide administration on macular edema: A systematic review and network meta-analysis
Source: PLoS One. 2025 Jan 24;20(1):e0317782. doi: 10.1371/journal.pone.0317782 (PMC11760001; doi:10.1371/journal.pone.0317782)
Supplement: S12 Table — Footnote: BCVA: Best corrected visual acuity; IVTA: Intravitreal injection triamcinolone; RITA: Retrobulbar injections triamcinolone; SCTA: Suprachoroidal triamcinolone; STiTA: Sub-Tenon’s infusion of triamcinolone; PLA: Placebo. (DOCX) [file pone.0317782.s020.docx]

## Supplementary Table 12. Exclusion of studies with non diabetic macular edema-Outcome: BCVA at the 12th week (Mean Difference; 95% confidence interval)

| **IVTA** |  |  |  |  |
| --- | --- | --- | --- | --- |
| -0.12 (-0.30, 0.05) | **PLA** |  |  |  |
| -0.09 (-0.30, 0.12) | -0.01 (-0.29, 0.27) | **RITA** |  |  |
| 0.05 (-0.17, 0.24) | -0.02 (-0.33, 0.29) | 0.13 (-0.17, 0.42) | **SCTA** |  |
| -0.05 (-0.17, 0.06) | 0.06 (-0.27, 0.39) | 0.04 (-0.20, 0.27) | -0.09 (-0.33, 0.14) | **STiTA** |

**Footnote:** BCVA: Best corrected visual acuity; IVTA: Intravitreal injection triamcinolone; RITA: Retrobulbar injections triamcinolone; SCTA: Suprachoroidal triamcinolone; STiTA: Sub-Tenon’s infusion of triamcinolone; PLA: Placebo.
