# Supplementary material for: Efficacy of different routes of triamcinolone acetonide administration on macular edema: A systematic review and network meta-analysis
Source: PLoS One. 2025 Jan 24;20(1):e0317782. doi: 10.1371/journal.pone.0317782 (PMC11760001; doi:10.1371/journal.pone.0317782)
Supplement: S13 Table — Footnote: BCVA: Best corrected visual acuity; IVTA: Intravitreal injection triamcinolone; RITA: Retrobulbar injections triamcinolone; SCTA: Suprachoroidal triamcinolone; STiTA: Sub-Tenon’s infusion of triamcinolone; PLA: Placebo. (DOCX) [file pone.0317782.s021.docx]

## Supplementary Table 13. Exclusion of studies with non diabetic macular edema-Outcome: BCVA at the 24th week (Mean Difference; 95% confidence interval)

| **IVTA** |  |  |  |  |
| --- | --- | --- | --- | --- |
| -0.08 (-0.21, 0.06) | **PLA** |  |  |  |
| -0.07 (-0.23, 0.09) | 0.01 (-0.15, 0.17) | **RITA** |  |  |
| 0.04 (-0.26, 0.34) | 0.12 (-0.21, 0.44) | 0.11 (-0.23, 0.44) | **SCTA** |  |
| -0.06 (-0.17, 0.06) | 0.02 (-0.16, 0.20) | 0.01 (-0.18, 0.21) | -0.1 (-0.42, 0.23) | **STiTA** |

**Footnote:** BCVA: Best corrected visual acuity; IVTA: Intravitreal injection triamcinolone; RITA: Retrobulbar injections triamcinolone; SCTA: Suprachoroidal triamcinolone; STiTA: Sub-Tenon’s infusion of triamcinolone; PLA: Placebo.
