# Supplementary material for: Efficacy of different routes of triamcinolone acetonide administration on macular edema: A systematic review and network meta-analysis
Source: PLoS One. 2025 Jan 24;20(1):e0317782. doi: 10.1371/journal.pone.0317782 (PMC11760001; doi:10.1371/journal.pone.0317782)
Supplement: S14 Table — Footnote: CMT: Central macular thickness; IVTA: Intravitreal injection triamcinolone; RITA: Retrobulbar injections triamcinolone; SCTA: Suprachoroidal triamcinolone; STiTA: Sub-Tenon’s infusion of triamcinolone; PLA: Placebo. (DOCX) [file pone.0317782.s022.docx]

## Supplementary Table 14. Exclusion of studies with non diabetic macular edema-Outcome: CMT at the 12th week (Mean Difference; 95% confidence interval)

| **IVTA** |  |  |  |  |
| --- | --- | --- | --- | --- |
| **-88.26 (-165.96, -13.30)** | **PLA** |  |  |  |
| -6.91 (-135.37, 121.04) | 81.31 (-46.09, 211.09) | **RITA** |  |  |
| 58.95 (-68.97, 187.30) | 147.17 (-0.10, 298.04) | 66.03 (-114.70, 247.18) | **SCTA** |  |
| -34.54 (-109.38, 33.14) | 53.87 (-43.75, 146.25) | -27.44 (-175.35, 111.76) | -93.48 (-243.97, 48.77) | **STiTA** |

**Footnote:** CMT: Central macular thickness; IVTA: Intravitreal injection triamcinolone; RITA: Retrobulbar injections triamcinolone; SCTA: Suprachoroidal triamcinolone; STiTA: Sub-Tenon’s infusion of triamcinolone; PLA: Placebo.
