# Supplementary material for: Efficacy of different routes of triamcinolone acetonide administration on macular edema: A systematic review and network meta-analysis
Source: PLoS One. 2025 Jan 24;20(1):e0317782. doi: 10.1371/journal.pone.0317782 (PMC11760001; doi:10.1371/journal.pone.0317782)
Supplement: S15 Table — Footnote: CMT: Central macular thickness; IVTA: Intravitreal injection triamcinolone; RITA: Retrobulbar injections triamcinolone; SCTA: Suprachoroidal triamcinolone; STiTA: Sub-Tenon’s infusion of triamcinolone; PLA: Placebo. (DOCX) [file pone.0317782.s023.docx]

## Supplementary Table 15. Exclusion of studies with non diabetic macular edema-Outcome: CMT at the 24th week (Mean Difference; 95% confidence interval)

| **IVTA** |  |  |  |  |
| --- | --- | --- | --- | --- |
| -32.8 (-105.44, 35.38) | **PLA** |  |  |  |
| -1.1 (-100.08, 98.47) | 31.74 (-65.97, 133.92) | **RITA** |  |  |
| 71.09 (-91.18, 232.4) | 104.31 (-70.9, 281.15) | 72.2 (-117.42, 259.87) | **SCTA** |  |
| -2.83 (-92.69, 68.85) | 30.56 (-85.01, 128.98) | -1.07 (-139.79, 114.39) | -74.36 (-262.72, 98.74) | **STiTA** |

**Footnote:** CMT: Central macular thickness; IVTA: Intravitreal injection triamcinolone; RITA: Retrobulbar injections triamcinolone; SCTA: Suprachoroidal triamcinolone; STiTA: Sub-Tenon’s infusion of triamcinolone; PLA: Placebo.
