# Supplementary material for: Efficacy of different routes of triamcinolone acetonide administration on macular edema: A systematic review and network meta-analysis
Source: PLoS One. 2025 Jan 24;20(1):e0317782. doi: 10.1371/journal.pone.0317782 (PMC11760001; doi:10.1371/journal.pone.0317782)
Supplement: S16 Table — Footnote: CMT: Central macular thickness; IVTA: Intravitreal injection triamcinolone; OFTA: Orbital floor triamcinolone; RITA: Retrobulbar injections triamcinolone; SCTA: Suprachoroidal triamcinolone; STiTA: Sub-Tenon’s infusion of triamcinolone; PLA: Placebo. (DOCX) [file pone.0317782.s024.docx]

## Supplementary Table 16. Exclusion of studies with fewer than 20 eyes-Outcome: CMT at the 12th week (Mean Difference; 95% confidence interval)

| **IVTA** |  |  |  |  |  |
| --- | --- | --- | --- | --- | --- |
| -75.89 (-248.20, 96.74) | **OFTA** |  |  |  |  |
| **-84.81 (-147.45, -24.27)** | -8.97 (-193, 173.11) | **PLA** |  |  |  |
| -34.13 (-128.97, 57.41) | 41.64 (-155.24, 236.15) | 50.59 (-49.30, 149.60) | **RITA** |  |  |
| 58.83 (-56.80, 174.51) | 134.3 (-72.96, 342.70) | **143.60(14.01, 275.51)** | 93.01 (-53.79, 242.86) | **SCTA** |  |
| -20.54 (-88.09, 44.56) | 55.23 (-130.9, 238.44) | 64.23 (-18.75, 147.43) | 13.55 (-98.34, 126.5) | -79.51 (-213.22, 52.73) | **STiTA** |

**Footnote:** CMT: Central macular thickness; IVTA: Intravitreal injection triamcinolone; OFTA: Orbital floor triamcinolone; RITA: Retrobulbar injections triamcinolone; SCTA: Suprachoroidal triamcinolone; STiTA: Sub-Tenon’s infusion of triamcinolone; PLA: Placebo.
