# Supplementary material for: Efficacy of different routes of triamcinolone acetonide administration on macular edema: A systematic review and network meta-analysis
Source: PLoS One. 2025 Jan 24;20(1):e0317782. doi: 10.1371/journal.pone.0317782 (PMC11760001; doi:10.1371/journal.pone.0317782)
Supplement: S17 Table — Footnote: CMT: Central macular thickness; IVTA: Intravitreal injection triamcinolone; OFTA: Orbital floor triamcinolone; RITA: Retrobulbar injections triamcinolone; SCTA: Suprachoroidal triamcinolone; STiTA: Sub-Tenon’s infusion of triamcinolone; PLA: Placebo. (DOCX) [file pone.0317782.s025.docx]

## Supplementary Table 17. Exclusion of studies with fewer than 20 eyes-Outcome: CMT at the 24th week (Mean Difference; 95% confidence interval)

| **IVTA** |  |  |  |  |  |
| --- | --- | --- | --- | --- | --- |
| -59.16 (-168.38, 50.23) | **OFTA** |  |  |  |  |
| -32.95 (-82.75, 11.79) | 25.81 (-95.11, 142.77) | **PLA** |  |  |  |
| -5.93 (-64.64, 50.09) | 53.07 (-71.23, 175.29) | 27.03 (-34.99, 91.59) | **RITA** |  |  |
| 70.71 (-61.75, 202.58) | 129.77 (-42.04, 300.92) | 104.2 (-35.11, 245.06) | 76.89 (-66.65, 221.02) | **SCTA** |  |
| 14.43 (-51.21, 77.94) | 73.49 (-54.29, 199.13) | 47.65 (-31.45, 128.06) | 20.58 (-66.14, 106.28) | -56.38 (-203.79, 90.15) | **STiTA** |

**Footnote:** CMT: Central macular thickness; IVTA: Intravitreal injection triamcinolone; OFTA: Orbital floor triamcinolone; RITA: Retrobulbar injections triamcinolone; SCTA: Suprachoroidal triamcinolone; STiTA: Sub-Tenon’s infusion of triamcinolone; PLA: Placebo.
