# Supplementary material for: Efficacy of different routes of triamcinolone acetonide administration on macular edema: A systematic review and network meta-analysis
Source: PLoS One. 2025 Jan 24;20(1):e0317782. doi: 10.1371/journal.pone.0317782 (PMC11760001; doi:10.1371/journal.pone.0317782)
Supplement: S18 Table — Footnote: BCVA: Best corrected visual acuity; IVTA: Intravitreal injection triamcinolone; OFTA: Orbital floor triamcinolone; RITA: Retrobulbar injections triamcinolone; SCTA: Suprachoroidal triamcinolone; STiTA: Sub-Tenon’s infusion of triamcinolone. (DOCX) [file pone.0317782.s026.docx]

## Supplementary Table 18. Exclusion of studies combined with laser therapy-Outcome: BCVA at the 12th week (Mean Difference; 95% confidence interval)

| **IVTA** |  |  |  |  |
| --- | --- | --- | --- | --- |
| -0.02 (-0.27, 0.23) | **OFTA** |  |  |  |
| 0 (-0.17, 0.17) | 0.02 (-0.29, 0.32) | **RITA** |  |  |
| 0.05 (-0.14, 0.22) | 0.07 (-0.25, 0.37) | 0.05 (-0.20, 0.29) | **SCTA** |  |
| -0.04 (-0.15, 0.05) | -0.02 (-0.3, 0.24) | -0.04 (-0.25, 0.15) | -0.1 (-0.3, 0.11) | **STiTA** |

**Footnote:** BCVA: Best corrected visual acuity; IVTA: Intravitreal injection triamcinolone; OFTA: Orbital floor triamcinolone; RITA: Retrobulbar injections triamcinolone; SCTA: Suprachoroidal triamcinolone; STiTA: Sub-Tenon’s infusion of triamcinolone;
