# Supplementary material for: Efficacy of different routes of triamcinolone acetonide administration on macular edema: A systematic review and network meta-analysis
Source: PLoS One. 2025 Jan 24;20(1):e0317782. doi: 10.1371/journal.pone.0317782 (PMC11760001; doi:10.1371/journal.pone.0317782)
Supplement: S19 Table — Footnote: BCVA: Best corrected visual acuity; IVTA: Intravitreal injection triamcinolone; OFTA: Orbital floor triamcinolone; RITA: Retrobulbar injections triamcinolone; SCTA: Suprachoroidal triamcinolone; STiTA: Sub-Tenon’s infusion of triamcinolone. (DOCX) [file pone.0317782.s027.docx]

## Supplementary Table 19. Exclusion of studies combined with laser therapy-Outcome: BCVA at the 24th week (Mean Difference; 95% confidence interval)

| **IVTA** |  |  |  |  |
| --- | --- | --- | --- | --- |
| -0.01 (-0.24, 0.22) | **OFTA** |  |  |  |
| -0.01 (-0.20, 0.18) | 0 (-0.30, 0.30) | **RITA** |  |  |
| 0.04 (-0.25, 0.33) | 0.05 (-0.32, 0.42) | 0.05 (-0.29, 0.39) | **SCTA** |  |
| -0.06 (-0.17, 0.05) | -0.05 (-0.30, 0.21) | -0.05 (-0.27, 0.17) | -0.1 0(-0.41, 0.21) | **STiTA** |

**Footnote:** BCVA: Best corrected visual acuity; IVTA: Intravitreal injection triamcinolone; OFTA: Orbital floor triamcinolone; RITA: Retrobulbar injections triamcinolone; SCTA: Suprachoroidal triamcinolone; STiTA: Sub-Tenon’s infusion of triamcinolone;
