# Supplementary material for: Efficacy of different routes of triamcinolone acetonide administration on macular edema: A systematic review and network meta-analysis
Source: PLoS One. 2025 Jan 24;20(1):e0317782. doi: 10.1371/journal.pone.0317782 (PMC11760001; doi:10.1371/journal.pone.0317782)
Supplement: S20 Table — Footnote: CMT: Central macular thickness; IVTA: Intravitreal injection triamcinolone; OFTA: Orbital floor triamcinolone; RITA: Retrobulbar injections triamcinolone; SCTA: Suprachoroidal triamcinolone; STiTA: Sub-Tenon’s infusion of triamcinolone; PLA: Placebo. (DOCX) [file pone.0317782.s028.docx]

## Supplementary Table 20. Exclusion of studies combined with laser therapy-Outcome: CMT at the 12th week (Mean Difference; 95% confidence interval)

| **IVTA** |  |  |  |  |  |
| --- | --- | --- | --- | --- | --- |
| -75.42 (-310.31, 160.05) | **OFTA** |  |  |  |  |
| -111.08 (-282.02, 55.79) | -35.94 (-327.86, 250.7) | **PLA** |  |  |  |
| -59.83 (-221.08, 97.63) | 15.38 (-269.60, 298.06) | 51.1 (-181.23, 284.55) | **RITA** |  |  |
| 58.98 (-102.72, 221.7) | 134.68 (-151.5, 419.57) | 170.36 (-61.93, 406.67) | 119.1 (-106.91, 347.77) | **SCTA** |  |
| -41.44 (-137.27, 46.13) | 33.89 (-222.57, 281.36) | 69.66 (-100.97, 235.36) | 18.41 (-167.78, 199.28) | -100.44 (-291.36, 81.58) | **STiTA** |

**Footnote:** CMT: Central macular thickness; IVTA: Intravitreal injection triamcinolone; OFTA: Orbital floor triamcinolone; RITA: Retrobulbar injections triamcinolone; SCTA: Suprachoroidal triamcinolone; STiTA: Sub-Tenon’s infusion of triamcinolone; PLA: Placebo.
