# Supplementary material for: Efficacy of different routes of triamcinolone acetonide administration on macular edema: A systematic review and network meta-analysis
Source: PLoS One. 2025 Jan 24;20(1):e0317782. doi: 10.1371/journal.pone.0317782 (PMC11760001; doi:10.1371/journal.pone.0317782)
Supplement: S21 Table — Footnote: CMT: Central macular thickness; IVTA: Intravitreal injection triamcinolone; OFTA: Orbital floor triamcinolone; RITA: Retrobulbar injections triamcinolone; SCTA: Suprachoroidal triamcinolone; STiTA: Sub-Tenon’s infusion of triamcinolone. (DOCX) [file pone.0317782.s029.docx]

## Supplementary Table 21. Exclusion of studies combined with laser therapy-Outcome: CMT at the 24th week (Mean Difference; 95% confidence interval)

| **IVTA** |  |  |  |  |  |
| --- | --- | --- | --- | --- | --- |
| -59.2 (-249.77, 131.57) | **OFTA** |  |  |  |  |
| -14.65 (-147.92, 115.05) | 44.06 (-188.09, 275.01) | **RITA** |  |  |  |
| 72.07 (-131.93, 275.56) | 131.34 (-147.35, 408.91) | 87.24 (-154.17, 329.51) | **SCTA** |  |  |
| -7.26 (-122.23, 84.42) | 52.29 (-177.78, 257.28) | 8.49 (-169.78, 163.14) | -79.49 (-318.6, 138.53) | **STiTA** |  |

**Footnote:** CMT: Central macular thickness; IVTA: Intravitreal injection triamcinolone; OFTA: Orbital floor triamcinolone; RITA: Retrobulbar injections triamcinolone; SCTA: Suprachoroidal triamcinolone; STiTA: Sub-Tenon’s infusion of triamcinolone;
