# Supplementary material for: Efficacy of different routes of triamcinolone acetonide administration on macular edema: A systematic review and network meta-analysis
Source: PLoS One. 2025 Jan 24;20(1):e0317782. doi: 10.1371/journal.pone.0317782 (PMC11760001; doi:10.1371/journal.pone.0317782)
Supplement: S22 Table — Footnote: BCVA: Best corrected visual acuity; IVTA: Intravitreal injection triamcinolone; OFTA: Orbital floor triamcinolone; RITA: Retrobulbar injections triamcinolone; SCTA: Suprachoroidal triamcinolone; STiTA: Sub-Tenon’s infusion of triamcinolone; PLA: Placebo. (DOCX) [file pone.0317782.s030.docx]

## Supplementary Table 22. Exclusion of studies with lost populations- Outcome: BCVA at the 12th week (Mean Difference; 95% confidence interval)

| **IVTA** |  |  |  |  |  |
| --- | --- | --- | --- | --- | --- |
| -0.02 (-0.31, 0.27) | **OFTA** |  |  |  |  |
| **-0.15 (-0.31, -0.01)** | -0.13 (-0.47, 0.19) | **PLA** |  |  |  |
| -0.08 (-0.25, 0.09) | -0.06 (-0.4, 0.28) | 0.07 (-0.11, 0.27) | **RITA** |  |  |
| 0.04 (-0.17, 0.25) | 0.06 (-0.3, 0.42) | 0.2 (-0.06, 0.46) | 0.13 (-0.15, 0.39) | **SCTA** |  |
| -0.04 (-0.18, 0.08) | -0.02 (-0.35, 0.29) | 0.11 (-0.09, 0.31) | 0.04 (-0.18, 0.25) | -0.09 (-0.33, 0.16) | **STiTA** |

**Footnote:** BCVA: Best corrected visual acuity; IVTA: Intravitreal injection triamcinolone; OFTA: Orbital floor triamcinolone; RITA: Retrobulbar injections triamcinolone; SCTA: Suprachoroidal triamcinolone; STiTA: Sub-Tenon’s infusion of triamcinolone; PLA: Placebo.
