# Supplementary material for: Efficacy of different routes of triamcinolone acetonide administration on macular edema: A systematic review and network meta-analysis
Source: PLoS One. 2025 Jan 24;20(1):e0317782. doi: 10.1371/journal.pone.0317782 (PMC11760001; doi:10.1371/journal.pone.0317782)
Supplement: S23 Table — Footnote: BCVA: Best corrected visual acuity; IVTA: Intravitreal injection triamcinolone; OFTA: Orbital floor triamcinolone; RITA: Retrobulbar injections triamcinolone; SCTA: Suprachoroidal triamcinolone; STiTA: Sub-Tenon’s infusion of triamcinolone; PLA: Placebo. (DOCX) [file pone.0317782.s031.docx]

## Supplementary Table 23. Exclusion of studies with lost populations- Outcome: BCVA at the 24th week (Mean Difference; 95% confidence interval)

| **IVTA** |  |  |  |  |  |
| --- | --- | --- | --- | --- | --- |
| -0.01 (-0.26, 0.24) | **OFTA** |  |  |  |  |
| -0.08 (-0.22, 0.06) | -0.07 (-0.35, 0.21) | **PLA** |  |  |  |
| -0.07 (-0.23, 0.09) | -0.06 (-0.35, 0.24) | 0.01 (-0.15, 0.17) | **RITA** |  |  |
| 0.04 (-0.26, 0.34) | 0.05 (-0.34, 0.44) | 0.12 (-0.21, 0.45) | 0.11 (-0.23, 0.45) | **SCTA** |  |
| -0.08 (-0.22, 0.07) | -0.07 (-0.35, 0.22) | -0.01 (-0.2, 0.2) | -0.02 (-0.22, 0.21) | -0.12 (-0.45, 0.22) | **STiTA** |

**Footnote:** BCVA: Best corrected visual acuity; IVTA: Intravitreal injection triamcinolone; OFTA: Orbital floor triamcinolone; RITA: Retrobulbar injections triamcinolone; SCTA: Suprachoroidal triamcinolone; STiTA: Sub-Tenon’s infusion of triamcinolone; PLA: Placebo.
