# Supplementary material for: Efficacy of different routes of triamcinolone acetonide administration on macular edema: A systematic review and network meta-analysis
Source: PLoS One. 2025 Jan 24;20(1):e0317782. doi: 10.1371/journal.pone.0317782 (PMC11760001; doi:10.1371/journal.pone.0317782)
Supplement: S24 Table — Footnote: CMT: Central macular thickness; IVTA: Intravitreal injection triamcinolone; OFTA: Orbital floor triamcinolone; RITA: Retrobulbar injections triamcinolone; SCTA: Suprachoroidal triamcinolone; STiTA: Sub-Tenon’s infusion of triamcinolone; PLA: Placebo. (DOCX) [file pone.0317782.s032.docx]

## Supplementary Table 24. Exclusion of studies with lost populations- Outcome: CMT at the 12th week (Mean Difference; 95% confidence interval)

| **IVTA** |  |  |  |  |  |
| --- | --- | --- | --- | --- | --- |
| -75.91 (-269.19, 118.6) | **OFTA** |  |  |  |  |
| -73.14 (-159.09, 8.92) | 2.53 (-210.44, 211.95) | **PLA** |  |  |  |
| -30.53 (-140.95, 75.45) | 45.27 (-178.61, 265.42) | 42.72 (-75.82, 160.39) | **RITA** |  |  |
| 59.39 (-72.15, 190.56) | 135.25 (-100.07, 369.28) | 132.67 (-21.28, 290.21) | 89.87 (-78.18, 262.08) | **SCTA** |  |
| -17 (-101.72, 57.27) | 58.87 (-157.01, 263.3) | 56.27 (-51.03, 156.25) | 13.58 (-120.4, 140.97) | -76.44 (-235.37, 71.44) | **STiTA** |

**Footnote:** CMT: Central macular thickness; IVTA: Intravitreal injection triamcinolone; OFTA: Orbital floor triamcinolone; RITA: Retrobulbar injections triamcinolone; SCTA: Suprachoroidal triamcinolone; STiTA: Sub-Tenon’s infusion of triamcinolone; PLA: Placebo.
