# Supplementary material for: Efficacy of different routes of triamcinolone acetonide administration on macular edema: A systematic review and network meta-analysis
Source: PLoS One. 2025 Jan 24;20(1):e0317782. doi: 10.1371/journal.pone.0317782 (PMC11760001; doi:10.1371/journal.pone.0317782)
Supplement: S25 Table — Footnote: CMT: Central macular thickness; IVTA: Intravitreal injection triamcinolone; OFTA: Orbital floor triamcinolone; RITA: Retrobulbar injections triamcinolone; SCTA: Suprachoroidal triamcinolone; STiTA: Sub-Tenon’s infusion of triamcinolone; PLA: Placebo. (DOCX) [file pone.0317782.s033.docx]

## Supplementary Table 25. Exclusion of studies with lost populations- Outcome: CMT at the 24th week (Mean Difference; 95% confidence interval)

| **IVTA** |  |  |  |  |  |
| --- | --- | --- | --- | --- | --- |
| -58.82 (-196.3, 80.19) | **OFTA** |  |  |  |  |
| -30.61 (-111.36, 41.7) | 27.82 (-133.98, 181.14) | **PLA** |  |  |  |
| -5.44 (-86.1, 71.19) | 53.33 (-107.86, 210.64) | 25.33 (-62.59, 117.98) | **RITA** |  |  |
| 71.44 (-84.85, 227.7) | 130.16 (-78.34, 338.09) | 103.02 (-68.2, 278.88) | 77.09 (-96.73, 252.37) | **SCTA** |  |
| 28.74 (-86.94, 107.65) | 87.55 (-101.08, 237.11) | 60.88 (-79.6, 164.04) | 35.05 (-108.78, 138.73) | -43.29 (-243.17, 124.49) | **STiTA** |

**Footnote:** CMT: Central macular thickness; IVTA: Intravitreal injection triamcinolone; OFTA: Orbital floor triamcinolone; RITA: Retrobulbar injections triamcinolone; SCTA: Suprachoroidal triamcinolone; STiTA: Sub-Tenon’s infusion of triamcinolone; PLA: Placebo.
