# Supplementary material for: Efficacy of different routes of triamcinolone acetonide administration on macular edema: A systematic review and network meta-analysis
Source: PLoS One. 2025 Jan 24;20(1):e0317782. doi: 10.1371/journal.pone.0317782 (PMC11760001; doi:10.1371/journal.pone.0317782)
Supplement: Attachment — Submitted filename: PONE-D-24-31871.docx [file pone.0317782.s034.docx]

**PONE-D-24-31871**

The manuscript discusses a network meta-analysis that evaluates the effectiveness of different ways of administering triamcinolone acetonide for treating macular edema. The study reviews randomized controlled trials (RCTs) to compare outcomes such as best corrected visual acuity (BCVA) and central macular thickness (CMT) associated with various administration methods, including intravitreal, suprachoroidal, and sub-Tenon’s routes. The findings suggest that intravitreal and suprachoroidal administration routes significantly improve BCVA and reduce CMT in the 12th week.

**Comments:**

**1. Title and Abstract**

The title accurately reflects the content and scope of the manuscript, focusing on the efficacy of different administration routes of triamcinolone acetonide for treating macular edema.

The abstract provides a clear and concise summary, highlighting the study's purpose, key findings, and significance. It effectively outlines the methodology and significant results, setting the stage for the detailed analysis in the manuscript.

**2. Introduction**

The introduction is well-structured and informative, providing sufficient background on macular edema and the role of triamcinolone acetonide.

**Literature Context:**

Relevant references are cited appropriately, establishing the study's importance and identifying gaps in the current literature regarding the comparative efficacy of different administration routes.

**3. Methodology**

The methods employed are appropriate for the research question. The systematic review and network meta-analysis design are well-suited to address the study's objectives.

The criteria for selecting studies are clearly defined and justified, ensuring the analysis includes relevant randomized controlled trials (RCTs).

**4. Results and Discussion**

Results are presented clearly, with effective use of tables and figures to complement the text.

The data analysis methods are appropriate, and the interpretation of statistical methods appears sound. However, the manuscript should clarify how varying dosages of triamcinolone across different RCTs might influence outcomes.

Findings are compared adequately with existing literature, and the discussion highlights the study's contributions to the field.

Limitations are acknowledged, including potential biases and the need for more high-quality RCTs. The impact of these limitations on findings is discussed.

**5. Considerations for Dosage and Adverse Effects**

The manuscript should address the variability in triamcinolone dosages used across different administration routes. Including a summary of the dosages from the included RCTs would enhance the manuscript’s robustness. Additionally, subgroup analyses based on dosage could help clarify how different dosages impact efficacy and safety.

A section summarizing the adverse effects of each administration route should be included. For instance, IVTA may lead to increased intraocular pressure and cataract formation, while SCTA could have risks like transient intraocular pressure changes. This information is crucial for a comprehensive risk-benefit analysis, guiding clinicians in selecting the most appropriate treatment route based on efficacy and safety.

**6. Conclusion**

The conclusion briefly summarizes the findings and implications. It should be emphasized that while SCTA and IVTA show promise in improving best corrected visual acuity (BCVA) and reducing central macular thickness (CMT) in the 12th week, the manuscript must cautiously address the limited differences observed in the 24th week. The discussion should encourage further large-scale, multi-center RCTs to validate findings and address identified limitations.

**7. Overall Quality**

The manuscript is generally well-written, though it contains minor grammatical errors that could be addressed through thorough proofreading.

Figures and tables are relevant, well-designed, and appropriately referenced in the text.

**8. Recommendations for Improvement**

Include a summary of dosages used in the included RCTs and consider subgroup analyses based on dosage.

Add a section discussing the adverse effects of each administration route, highlighting their implications for patient safety and treatment preference.

Enhance the clarity and conciseness of some sections, along with a thorough review for grammatical accuracy.

9. **Reviewer’s conclusion**

Overall, the manuscript presents a valuable contribution to the field, with robust findings from a well-conducted meta-analysis. It is suitable for publication, but minor revisions are needed to enhance clarity and comprehensiveness. Also, adding a section discussing the adverse effects and a summary of the doses used across the RCTs would provide additional value to the manuscript.
