# Supplementary material for: Efficacy of different routes of triamcinolone acetonide administration on macular edema: A systematic review and network meta-analysis
Source: PLoS One. 2025 Jan 24;20(1):e0317782. doi: 10.1371/journal.pone.0317782 (PMC11760001; doi:10.1371/journal.pone.0317782)
Supplement: Attachment — Submitted filename: Response to Reviewers.docx [file pone.0317782.s035.docx]

**Response to** **Reviewers**

Dear Reviewers and editor,

Thank you for your insightful comments on the manuscript. We have thoroughly reviewed your suggestions and have implemented the following revisions accordingly. We anticipate the publication of the paper in ***PLOS ONE*** with great interest.

1. The authors referred to the stylistic requirements of PLOS ONE and reconfirmed manuscript formatting, including file naming.
2. The authors agree that PLOS ONE has specific guidelines for submitted code shares.
3. The authors have updated the Funding Statement in the manuscript as requested, incorporating a declaration that "no additional external funding was received for this study." Furthermore, we have incorporated the revised Funding Statement into the cover letter.
4. The authors add the captions of the Supporting Information file at the end of the manuscript and update the corresponding textual citations in accordance with the Supporting Information guidelines.
5. The authors reconfirm the reference list, and we did not cite the retracted papers.
6. In the "Characteristics of eligible studies" section, the authors provide a detailed description of a summary of the doses used of triamcinolone acetonide (TA) in the included randomized controlled trials (RCTs). Given that the dosage of TA varies depending on the route of administration, the RCTs included in the analysis using the two statistical methods remains same, provided that different routes of administration and dosages are examined independently. When attempting to conduct a subgroup analysis of varying doses administered via the same route, we were unable to proceed due to the limited number of studies available in this area. This limitation is addressed in the discussion section of this article. However, we intend to revisit this analysis should more relevant studies become available in the future. Many thanks to the reviewers for pointing out the specific direction for our future work.
7. In response to the reviewers' recommendations, the author incorporated an additional paragraph into the discussion section of the article to elaborate on and analyze the adverse reactions associated with each administration route of TA, drawing upon existing literature. Furthermore, we express our gratitude to the reviewers for their insights, which will guide us in undertaking more comprehensive investigations into the safety of various drug delivery routes in subsequent studies.

Sincerely,

Na Su

Department of Pharmacy, West China Hospital, Sichuan University, Chengdu, China.
